# Silver and Cyanine Staining of Oligonucleotides in Polyacrylamide Gel

**DOI:** 10.1371/journal.pone.0144422

**Published:** 2015-12-09

**Authors:** Weizhong Tang, Huafu Zhou, Wei Li

**Affiliations:** 1 Department of Colorectal Surgery, The First Affiliated Hospital of Guangxi Medical University, 6 Shuangyong Road, Nanning, Guangxi 530021, China; 2 Department of Cardiothoracic Surgery, The First Affiliated Hospital of Guangxi Medical University, 6 Shuangyong Road, Nanning, Guangxi 530021, China; 3 Medical Scientific Research Center, Guangxi Medical University, 22 Shuangyong Road, Nanning, Guangxi 530021, China; St. Georges University of London, UNITED KINGDOM

## Abstract

To explore why some oligonucleotides in denaturing polyacrylamide gel could not be silver-stained, 134 different oligonucleotides were analyzed using denaturing polyacrylamide gel electrophoresis stained with silver and asymmetric cyanine. As a result, we found that the sensitivity of oligos (dA), (dC), (dG) and (dT) to silver staining could be ranged as (dA) > (dG) > (dC) > (dT) from high to low. It was unexpected that oligo (dT) was hard to be silver-stained. Moreover, the silver staining of an oligonucleotide containing base T could be partially or completely inhibited by base T. The inhibition of silver staining by base T was a competitive inhibition which could be affected by the amounts of the argyrophil nucleobase and base T, the cis-distance between the argyrophil nucleobase and base T, and the gel concentration. The changes of the intensity of an oligonucleotide band caused by the changes of DNA base composition were diverse and interesting. The intensity of some oligonucleotide bands would significantly change when the changes of DNA base composition accumulated to a certain extent (usually ≥ 4 nt). The sensitivity of cyanine staining of ≤ 11-nt long oligonucleotides could be enhanced about 250-fold by fixing the gels with methanol fixing solution.

## Introduction

The integrity of an oligonucleotide (oligo) is important for many experiments (such as PCR, reverse transcription and RNAi, etc) and able to be examined by using agarose gel electrophoresis or denaturing polyacrylamide gel electrophoresis (PAGE). DNA in a PAGE gel can be visualized by using radioisotopic and non-radioisotopic methods. Radioisotopic method is highly sensitive, and almost limitless on the minimum length of DNA fragments. But it is harmful to the users and difficult to be handled, and therefore, has been almost completely replaced by non-radioisotopic methods such as staining with ethidium bromide (EB), asymmetric cyanine dyes, and silver, etc. EB staining is cheaper than the staining with silver or asymmetric cyanine. However, since EB is a strong mutagen and carcinogen [1–3], it is being gradually replaced with asymmetric cyanine dyes, etc.

Asymmetric cyanine dyes include SYBR Green I, SYBR Green II and SYBR Gold, etc. SYBR Gold is one of the best asymmetric cyanine dyes and exhibits a high affinity to nucleic acids, a > 1000-fold fluorescence enhancement and a quantum yield of ~0.6 upon binding [4], and therefore can be used in the visualization of double-stranded DNA (dsDNA), single-stranded DNA (ssDNA) and RNA [4] [5]. SYBR Green I is more sensitive to dsDNA than to RNA and ssDNA. Less than 20 pg of dsDNA in a single band can be detected by using SYBR Green I (Molecular Probes: Product Information. http://www.mobitec.com/probes/docs/media/pis/mp07567.pdf.). SYBR Green II can be applied for detecting dsDNA, ssDNA and RNA. But it exhibits a higher quantum yield when bound to RNA and ssDNA than bound to dsDNA (Molecular Probes: Product Information. http://www.mobitec.de/probes/docs/media/pis/mp07568.pdf.).

The sensitivity of DNA silver staining is as high as that of radioisotope [6], 1000 times higher than EB staining. In 1979, Merril and colleagues first introduced silver staining for the visualization of protein in polyacrylamide gel [7]. Since then, many silver staining methods have been developed for the detection of protein and DNA in PAGE gel. These methods can be classified into two groups, one of them is derived from histology [8–11], and the other one is derived from photochemistry [12–15]. In 1981, a faster, more reliable, and very sensitive silver staining method was introduced to detect protein by Merril et al [16]. In 1991 and 1993, Bassam and colleagues introduced another DNA silver staining method based on a photochemically derived staining procedure [17] [18]. Bassam’s method works well for the detection of long DNA fragments. However, we found that it did not work well for some short DNA fragments (< 80 bp) when we detected thalassemia mutations by using single strand conformation polymorphism (SSCP) [19]. In 2012, we found that some oligos could not be silver-stained in denaturing PAGE gels [20]. Here we analyzed 134 different oligos in order to explore why some oligos could not be silver-stained.

## Materials and Methods

### Syntheses of deoxy-oligos

134 different deoxy-oligos were synthesized by Sangon (China). Their sequences were listed in S1 Table. Most of these oligos were consisted of 2 types of nucleobases, and most of them could be arranged into different oligo-sets in which one type of nucleobases was gradually substituted by another type of nucleobases from 5′-end to 3′-end, or vice versa. For example, in oligo-set (A_7_C, A_6_C_2_, A_5_C_3_, A_4_C_4_, A_3_C_5_, A_2_C_6_, and AC_7_), base A was gradually substituted by base C from 5′-end to 3′-end. We called this oligo-set as oligo-set (A-C). Following this principle, other oligo-sets were called as oligo-sets (A-G), (A-T), (C-A), etc. Some oligos were consisted of only one type of nucleobases, such as oligos (dA), (dC), (dG), and (dT). The other oligos were consisted of 3–4 types of nucleobases, such as oligos (ACG)_3_ and (ACGT)_2_. The lengths of oligos were 5–59 nt long. 5-nt long oligos were the shortest oligos commercially available for us. > 5-nt long oligo (dG) was not available for us because it was difficult to be synthesized. After syntheses, the oligos (dA), (dC), (dG) and (dT) which were ≤ 8 nt long, such as oligos A_5_, C_5_, G_5_ and T_5_, were purified by using High Performance Liquid Chromatography (HPLC), while the others were purified by using High Affinity Purification (HAP) method. Additionally, some of oligos A_8_, C_8_, G_8_ and T_8_ were also purified by using HAP. Each oligo was dissolved into a 100–1000 μM solution with sterile ddH_2_O. Their actual concentrations were determined using NanoDrop 2000 (Thermo Scientific, USA) and stored at -20°C.

### PAGE

Mini gels (10 × 10 × 0.03 cm^3^) were prepared with shark tooth combs. The electrophoretic unit was SE260 (Hoefer, USA). The gel concentrations were 15%, 20%, 25%, 30%, 35%, 40%, and 45%. Acrylamide/bisacrylamide = 19/1. The urea concentrations in 15–35%, 40%, and 45% denaturing gels were 7, 6 and 3.5 M, respectively. Gel buffer and tank buffer were 1× and 0.5 × TBE, respectively. The sample volume was 2.5–6 μl. 0.01–10 μg of an oligo were mixed with 2 μl of formamide loading dye containing 98% (v/v) formamide, 10 mM EDTA (pH 8.0) and 0.05% (w/v) bromophenol blue, denatured at 95°C for 30 seconds, cooled on ice, and then loaded to the gels. Electrophoreses were run at room temperature. The electrophoretic voltages and times for different concentration gels were: 15%, 200 V, 1 hr; 20%, 300 V, 1 hr; 25%, 400 V, 1 hr; 30%, 500 V, 1.7–2.5 hrs; 35%, 600 V, 2–2.5 hrs; 40%, 700 V, 3.5–4 hrs; 45%, 800 V, 4.5–5 hrs.

### Silver staining

Since some short DNA fragments were difficult to be silver-stained by using Bassam’s method [19], here the oligos were silver-stained following Beidler’s method which could detect 10-20-nt long oligos [12], but some modifications were made. Briefly, a gel was fixed for 1.5–2 hrs with 50 ml of methanol fixing solution containing 50% (v/v) methanol, and 10% (v/v) glacial acetate acid, washed 3 times (3 min each) with 200 ml of deionized water, stained for 30 min with 30 ml of 0.1% AgNO_3_ containing 45 μl of 37–40% formaldehyde, developed with 30 ml of 3% Na_2_CO_3_ containing 45 μl of 37–40% formaldehyde and 30 μl of 2 mg/ml Na_2_S_2_O_3_ for about 3 min until DNA bands were visible clearly, and then, stopped the development with 20 ml of 10% acetic acid. The oligos (dA), (dC), (dG) and (dT) which were < 8 nt long were fixed, washed, and stained at 4°C, but developed at room temperature (16–29°C).

### Cyanine staining

After electrophoreses, the gels were fixed using methanol fixing solution as mentioned above, washed 3 times (3 min each) with 200 ml of deionized water, stained for 30–60 min with 10 ml of 10,000-fold-dilution solution of 10,000 × SYBR Green II RNA gel stain (SGRGS, Solarbio, China) or 10 ml of 5000-fold-dilution solution of Green-DNA Dye (GDD, Sangon, China) diluted with deionized water following the manufacturer’s instruction, and then the oligo bands were viewed using Gel Doc^™^ XR+ System (Bio-Rad, USA). According to the manufacturer’s instruction, SGRGS is 10,000 × concentration of SYBR Green II (its purity is ≥ 98%) in DMSO, whereas GDD is 5,000 × concentration of SYBR Green I (its purity is ≥ 99%) in DMSO. To see whether oligo A_11_ could be stained with GDD in 15% denaturing gel without the gel-fixing, 2–10 μg per well of oligo A_11_ were run electrophoresis, and then, the gel were washed 2 times (3 min each) with 200 ml of 0.5 × TBE, stained with 10 ml of 5000-fold-dilution solution of GDD (diluted with 0.5 × TBE) without the gel-fixing. The oligos (dA), (dC), (dG) and (dT) which were < 8nt long were fixed and washed at 4°C, and then stained with GDD at 4°C.

### Estimation of the integrated densities

Integrated density (IntDen) of oligo bands stained with silver or asymmetric cyanine was estimated by using Image J (NIH, USA). The intensity of an oligo band was represented with its Δ IntDen. Δ IntDen = (IntDen of an oligo band)—(IntDen of an equivalent-area background).

## Results

### Silver staining

**Fig 1:**
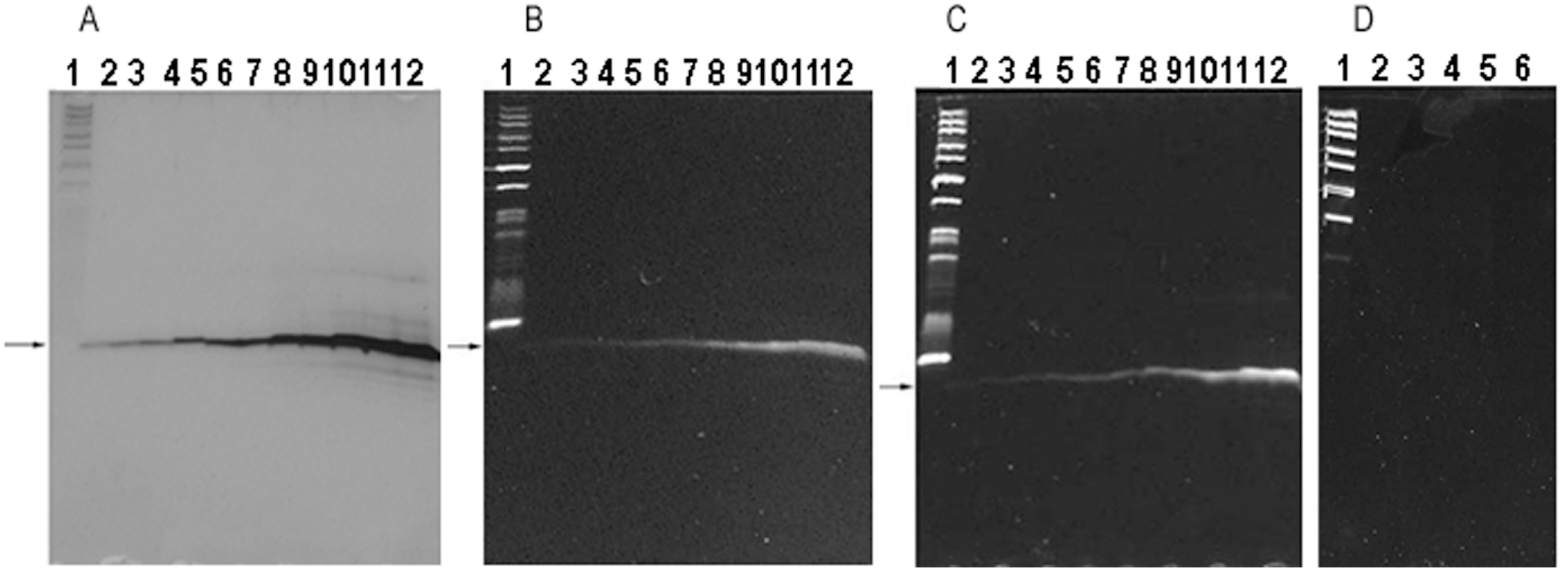
Oligo A_11_ in 15% denaturing PAGE gels. DNA marker was DNA marker 1 (GeneRuler^™^ Ultra low Range DNA ladder, Fermentas, Lithuania). The arrows indicate the specific oligo bands. (A), (B) and (C) The gels were stained with silver, SGRGS and GDD, respectively. Lane 1 was DNA marker 1. Lanes 2–12: 0.01, 0.02, 0.04, 0.06, 0.08, 0.1, 0.2, 0.4, 0.6, 0.8 and 1μg of oligo A_11_. (D) The gel was directly stained with GDD without fixing the gel before staining. Lane 1 was DNA marker 1. Lanes 2–6: 2, 4, 6, 8 and 10 μg of oligo A_11_.

1. Most of the oligos could be silver-stained in denaturing gels. S1A1–S1K1 Fig show the oligo bands of 8-nt long oligo-sets in 35% denaturing gels. Oligo-sets (C-T), (T-C), (G-T) and (T-G) were difficult to be silver-stained in 15–40% denaturing gels (S1H1–S1K1 Fig and S2A1–S2O1 Fig). The oligo bands of 0.01–1μg of oligo A_11_ were shown in Fig 1A. The Δ IntDen of all the oligos silver-stained was listed in Table 1. If an oligo could be silver-stained, its band should be darker than the background. Therefore the Δ IntDen of an oligo band silver-stained should be a negative number. The higher the absolute value of Δ IntDen was, the stronger the oligo band was. Δ IntDen (silver staining) changes from 0 to -631928 in Table 1. When the absolute value of Δ IntDen of an oligo band was < 8000, the oligo band was pretty weak. In addition, some oligo bands of oligo-sets (C-T), (T-C), (G-T) and (T-G) were so hazy that they looked like mist but not a band (S1H1–S1K1 Fig and S2A1–S2O1 Fig). The Δ IntDen from these hazy oligo bands was recorded in italics in Table 1. The Δ IntDen of 0.01–1μg of oligo A_11_ silver-stained was listed in S2 Table.

**Table 1:** Δ IntDen of oligo stained with silver and cyanine (silver staining/cyanine staining).

Oligos	LAO	15% gel	20% gel	25% gel	30% gel	35% gel	40% gel	45% gel
A_5_	1	-290639/Z	-187187/Z	-189125/Z	-245141/Z	-135460/*334*	-235696/Z	-171396/Z
A_6_	1	-220934/*84*	-185585/*179*	-160334/*773*	-207725/*351*	-93760/*606*	-204940/Z	-135103/Z
A_7_	1	-319500/2695	-300809/5294	-217587/9999	-247365/6538	-183903/7005	-270811/5953	-236730/5266
A_8_	1	-460362/40457	-398137/17106	-356531/18423	-415641/18434	-343041/9734	-427942/23869	
A_9_	0.056	-168363/4127	-179836/2956	-137205/7095	-149136/4046	-157691/5106	-160359/5668	
C_5_	1	Z/1309	Z/2791	Z/5708	Z/3679	Z/2406	Z/2395	Z/2025
C_6_	1	Z/4634	*-5717*/5179	*-3513*/10541	Z/5233	Z/4563	Z/4925	Z/3206
C_7_	1	*-22040*/13178	*-9181*/13149	*-10841*/13005	*-5312*/7141	*-2505*/8144	Z/6773	Z/7824
C_8_	1	-29096/18318	-37681/15455	-41273/11417	-32824/14009	-33367/5631	-52557/28102	
C_9_	1	-60702/30329	-43744/10200	-52617/17038	-83187/8191	-93880/1517	-94768/3863	
G_5_	1	-80833/24629	-56030/26266	-46894/33756	-47092/17905	-46209/12914	-30667/10313	-40639/3455
T_5_	1	Z/1712	Z/1165	Z/1497	Z/1310	Z/1807	Z/1268	Z/1221
T_6_	1	Z/1941	Z/3339	Z/1327	Z/1269	Z/1509	Z/1818	Z/404
T_7_	1	Z/5219	Z/9236	Z/5545	Z/3863	Z/1822	Z/3084	Z/1011
T_8_	1	Z/5996	Z/17897	Z/9432	Z/11981	Z/4959	Z/16332	
T_9_	1	Z/149679						
T_10_	1	Z/100002						
T_14_	1	Z/74666	Z/	Z/	Z/	Z/	Z/	
T_29_	1	Z/86418	Z/	Z/	Z/	Z/	Z/	
T_31_	1	Z/85809	Z/	Z/	Z/	Z/	Z/	
T_40_	1	Z/35075	Z/	Z/	Z/	Z/	Z/	
T_45_	1	Z/43245	Z/	Z/	Z/	Z/	Z/	
T_50_	1	Z/55001	Z/	Z/	Z/	Z/	Z/	
T_59_	1	Z/22758	Z/	Z/	Z/	Z/	Z/	
AC_7_	1	-24384/3213	-22355/2821	-17402/2979	-27162/3625	-7129/9517	-16795/17111	
A_2_C_6_	1	-39203/Z	-38815/Z	-25974/756	-43420/Z	-17407/3471	-23046/5829	
A_3_C_5_	1	-29898/Z	-27248/Z	-33296/1533	-49672/Z	-109351/3080	-170561/2511	
A_4_C_4_	1	-177512/Z	-248651/Z	-395269/Z	-294877/Z	-255108/2284	-312143/280	
A_5_C_3_	1	-185324/Z	-276780/Z	-455022/Z	-358302/Z	-348553/2248	-315887/238	
A_6_C_2_	1	-134088/Z	-285650/Z	-586118/Z	-348959/Z	-370325/1629	-390412/275	
A_7_C	1	-113425/Z	-432372/Z	-533629/Z	-384320/Z	-391803/*3651*	-443360/542	
(AG_3_)_2_	1	-194078/328374	-272463/332664	-177192/290908	-211766/294286	-198540/259735	-212867/400291	
A_2_G_3_AG_2_	1	-212921/138258	-247677/89297	-197399/129619	-339751/106581	-289400/101090	-285124/198033	
A_3_G_5_	1	-292722/184086	-231076/138591	-210724/149031	-420211/161398	-287267/138337	-282394/232837	
A_4_G_4_	1	-257040/53666	-399854/47181	-368319/74173	-487714/48400	-353572/66331	-306630/100259	
A_5_G_3_	1	-156316/5815	-361860/6553	-321511/16087	-547648/13851	-330465/19983	-339425/42752	
A_6_G_2_	1	-133728/Z	-172081/Z	-318397/3339	-484125/5176	-322757/4031	-291219/9370	
A_7_G	1	-76471/Z	-158963/Z	-272776/2991	-417299/1533	-218543/5783	-263020/9796	
AT_7_	1	Z/2868	Z/24919	Z/43637	Z/40053	Z/66475	Z/57597	
A_2_T_6_	1	Z/4359	Z/30056	Z/77090	Z/49315	Z/130764	Z/75307	
A_3_T_5_	1	Z/3302	Z/32314	Z /67109	Z/36054	Z/180475	Z/61470	
A_4_T_4_	1	Z/2863	Z/21519	-17854/28091	-15937/24172	-70968/322290	-21425/79798	
A_5_T_3_	1	Z/20645	-28303/22650	-156942/74702	-106913/20577	-267727/11951	-236483/22656	
A_6_T_2_	1	Z/Z	Z/7206	-45253/20800	-39991/5341	-248989/*3749*	-304766/8390	
A_7_T	1	-410536/Z	-169360/Z	-377590/Z	-373380/*4285*	-435014/*2745*	-366032/*2594*	
AT_9_	1	Z/38205						
A_2_T_8_	1	Z/55608						
A_3_T_7_	1	Z/58088						
A_4_T_6_	1	Z/37433						
A_5_T_5_	1	Z/21974						
A_6_T_4_	1	Z/8443						
A_7_T_3_	1	Z/*2974*						
A_8_T_2_	1	-213938/14157						
A_9_T	1	-534760/10429						
CA_7_	1	-546349/20680	-387482/39971	-410948/38295	-341779/46477	-425344/32662	-401824/49079	
C_2_A_6_	1	-89230/Z	-245606/Z	-238242/2747	-318917/1344	-370313/1990	-445700/5919	
C_3_A_5_	1	-21713/Z	-227636/Z	-228842/*2259*	-294898/Z	-355360/Z	-397948/4519	
C_4_A_4_	1	-10987/Z	-49683/Z	-75404/4431	-124145/Z	-278525/1447	-283463/4914	
C_5_A_3_	1	-21438/Z	-32212/*3952*	-47940/6163	-59122/3240	-118275/2650	-185438/9410	
C_6_A_2_	1	-29476/*2141*	-38039/5960	-56026/7498	-40781/7775	-18909/12744	-111638/9828	
C_7_A	1	-23223/4869	-17821/13154	-20486/12330	-12354/14721	-11896/23440	-26954/17942	
(CG_3_)_2_	1	-93960/30969	-49200/56839	-97317/17561	-112903/33808	-207072/42207	-241842/31103	
CGCG_5_	1	-48229/40608	-44324/65244	-54750/38136	-79277/44645	-169348/66508	-193632/31233	
C_3_G_5_	1	-37678/34466	-25655/81569	-46817/36117	-42674/61635	-113813/99121	-135106/52564	
C_4_G_4_	1	-51908/19170	-43570/88758	-58924/12948	-57398/212711	-100020/343960	-130577/232851	
C_5_G_3_	1	-63448/45189	-35351/14353	-41211/7331	-32730/46911	-55520/107054	-94089/82712	
C_6_G_2_	1	-43916/55788	-26317/15282	-18357/13515	-16619/11047	-16668/23128	-45716/16303	
C_7_G	1	-47219/13495	-25232/4681	-20688/20031	-19568/10083	-29713/6450	-45494/6567	
CT_7_	1	Z/21215	*-11889*/18099	*-10295*/20925	*-10101*/16317	*-8437*/43591	*-3225*/13393	
C_2_T_6_	1	Z/Z	*-2799*/*9181*	*-4871*/7759	*-10386*/*10363*	*-3783*/11849	Z/6037	
C_3_T_5_	1	Z/Z	Z/Z	*-1961*/*4491*	Z/*2814*	*-2526*/9142	Z/2535	
C_4_T_4_	1	Z/Z	Z/Z	Z/*3750*	Z/*3561*	Z/7166	Z/1271	
C_5_T_3_	1	Z/Z	Z/Z	Z/3156	Z/*2979*	Z/5412	Z/2820	
C_6_T_2_	1	Z/Z	Z/6795	Z/2768	Z/3536	Z/16852	Z/3063	
C_7_T	1	Z/2632	Z/5423	Z/9867	Z/7169	Z/20682	Z/5293	
GA_7_	1	-631928/*1153*	-406687/11185	-195875/15752	-225181/14683	-327728/23053	-359499/6640	
G_2_A_6_	1	-354099/*407*	-402296/4800	-282830/5094	-307206/*1457*	-290135/5225	-392602/4942	
G_3_A_5_	1	-492379/*1606*	-376484/25420	-378090/11788	-375634/*3023*	-342148/9968	-369196/11600	
G_3_AGA_3_	1	-409131/*6165*	-314361/36033	-434623/27588	-380015/3420	-316613/10620	-330208/27848	
G_3_AG_2_A_2_	1	-204385/44642	-209908/65089	-345849/53708	-385985/10164	-283097/17938	-337553/33364	
(G_3_A)_2_	1	-400311/119157	-115758/155567	-167863/144960	-268010/60009	-202380/77855	-273226/62140	
G_3_AG_4_	1	-323726/176673	-83365/258571	-89412/228151	-171407/104655	-110926/235020	-230267/172339	
G_5_AG_2_	1	-199265/473566	-93499/445014	-118789/388939	-181748/157915	-182112/293541	-314684/332534	
GC_7_	1	-82196/2206	-62104/6792	-60368/6550	-46279/10656	-66275/12056	-51597/7362	
G_2_C_6_	1	-25971/1643	-30832/4841	-42537/2931	-30052/1701	-44306/9450	-49134/6367	
G_3_C_5_	1	-11500/5141	-12387/3050	-29878/8142	-31168/1386	-30526/12582	-60612/19027	
G_4_C_4_	1	-23722/33818	-26765/46424	-55558/19707	-60476/7896	-53828/35042	-124847/39399	
G_5_C_3_	1	-22750/27427	-29129/107150	-99452/49351	-63001/45759	-73689/74239	-157382/64390	
G_5_C_2_G	1	-57057/41419	-67441/104267	-147617/48739	-103210/54435	-133668/75931	-176645/75947	
G_5_CG_2_	1	-172305/101374	-210880/170455	-352316/92396	-275387/69319	-285852/110059	-290623/114190	
GT_7_	1	*-14033*/22721	*-12042*/66894	*-7911*/36214	*-10482*/81608	*-14953*/51227	*-15398*/34617	
G_2_T_6_	1	Z/17375	*-19329*/50950	*-4522*/41092	*-11746*/62994	-15102/57906	*-30412*/26009	
G_3_T_5_	1	Z/50713	Z/62211	Z/68029	Z/107727	*-7106*/95263	Z/29445	
G_4_T_4_	1	Z/238232	Z/117793	Z/147978	Z/146353	Z/158711	Z/61212	
G_5_T_3_	1	Z/315911	Z/175604	Z/202830	Z/281754	Z/282310	-4321/62614	
G_5_TGT	1	*-11591*/340940	*-3978*/283662	*-4493*/268019	*-12073*/302744	*-1006*/313187	*-22664*/135722	
G_5_TG_2_	1	-60022/381543	-45723/321726	-28169/312515	-64687/380047	-64022/370264	-138461/223595	
G_2_TG_5_	1	-56935/289467	-45329/432604	-100330/254736	-57350/277295	-113640/426941	-170770/346485	
TA_7_	1	-16657/5888	-392209/14003	-345235/15655	-414597/6467	-372122/15582	-383120/10408	
T_2_A_6_	1	Z/*3774*	-119506/8392	-193556/13444	-322869/*3833*	-374122/19906	-355530/5760	
T_3_A_5_	1	Z/13400	Z/17209	-50918/27924	-51212/*5452*	-215828/42500	-249363/10681	
T_4_A_4_	1	Z/39038	Z/42094	Z/45983	Z/26098	Z/80444	Z/20531	
T_5_A_3_	1	Z/39140	Z/84056	Z/96169	Z/33072	Z/144694	Z/50288	
T_6_A_2_	1	Z/67498	Z/92316	Z/119375	Z/55652	Z/123473	Z/59612	
T_7_A	1	Z/74616	Z/66967	Z/83254	Z/50565	Z/92707	Z/41997	
TC_7_	1	Z/1595	Z/4561	Z/36498	Z/8413	*-847*/3166	Z/30395	
T_2_C_6_	1	Z/1247	Z/1281	Z/7454	Z/7508	*-3195*/2006	Z/19305	
T_3_C_5_	1	Z/*1372*	Z/1625	Z/2922	Z/4331	*-2911*/1662	Z/11535	
T_4_C_4_	1	Z/*1105*	Z/*680*	Z/5208	Z/*4618*	*-3397*/1794	*-5332*/8613	
T_5_C_3_	1	Z/*3123*	Z/*1176*	Z/4509	Z/*3007*	*-11370*/3698	*-4513*/5157	
T_6_C_2_	1	Z/*5762*	Z/3600	Z/13433	Z/*7362*	*-21214*/6680	*-5780*/14190	
T_7_C	1	Z/12676	Z/16022	Z/37819	Z/17244	*-25555*/12273	*-8928*/30253	
TGTG_5_	1	*-2033*/195744	*-1864*/323468	-18925/187306	-12743/198163	-32588/283188	-20175/316664	
T_3_G_5_	1	Z/176125	Z/242385	Z/155731	*-13500*/127599	*-6845*/224997	*-7785*/270954	
T_4_G_4_	1	Z/146364	Z/187731	Z/107129	*-8072*/109261	*-6163*/173686	Z/192689	
T_5_G_3_	1	Z/92292	Z/90078	Z/65392	*-11264*/82660	*-5044*/117969	Z/126022	
T_6_G_2_	1	*-4410*/54000	*-981*/64827	*-8852*/52954	*-15322*/75944	*-16659*/90696	*-11919*/106925	
T_7_G	1	*-6653*/36774	*-8203*/48257	*-1379*/52662	*-19309*/66230	*-19995*/77047	*-23620*/104628	
(ACG)_3_	1	-25667/183833						
(ACGT)_2_	1	Z/211587	Z/	Z/	Z/	Z/	-5313/	
(A_5_T)_2_	1	-108075/221489						
A_7_T_20_	1	Z/						
A_8_T_19_	0.75	Z/101653						
A_8_T_19_	1	Z/						
A_9_T_18_	0.75	Z/109602						
A_9_T_18_	1	Z/						
A_10_T_6_	1	-165478/130767						
A_10_T_8_	1	-19361/39600						
A_10_T_10_	1	-48185/39764	-76450/	-116735/	-73404/			
A_11_T_11_	1	-137444/330065	-123935/	-145830/	-101668/			
A_12_T_12_	1	-213106/562867	-194751/	-183434/	-205620/			
(TA)_12_	1	*-26575*/203644	-56758/	-54558/	-41045/			
A_14_T_10_	1	-266929/278990						
A_15_T_15_	1	-218771/						
A_17_T_10_	1	-343222/264318						
A_20_T_20_	1	-122634/						

LAO = Loading amount of oligos (μg). Z = Zero, representing that the oligo band is invisible. Blank = untested. The numbers in italics represent the Δ IntDen from the hazy oligo bands.

**Fig 2:**
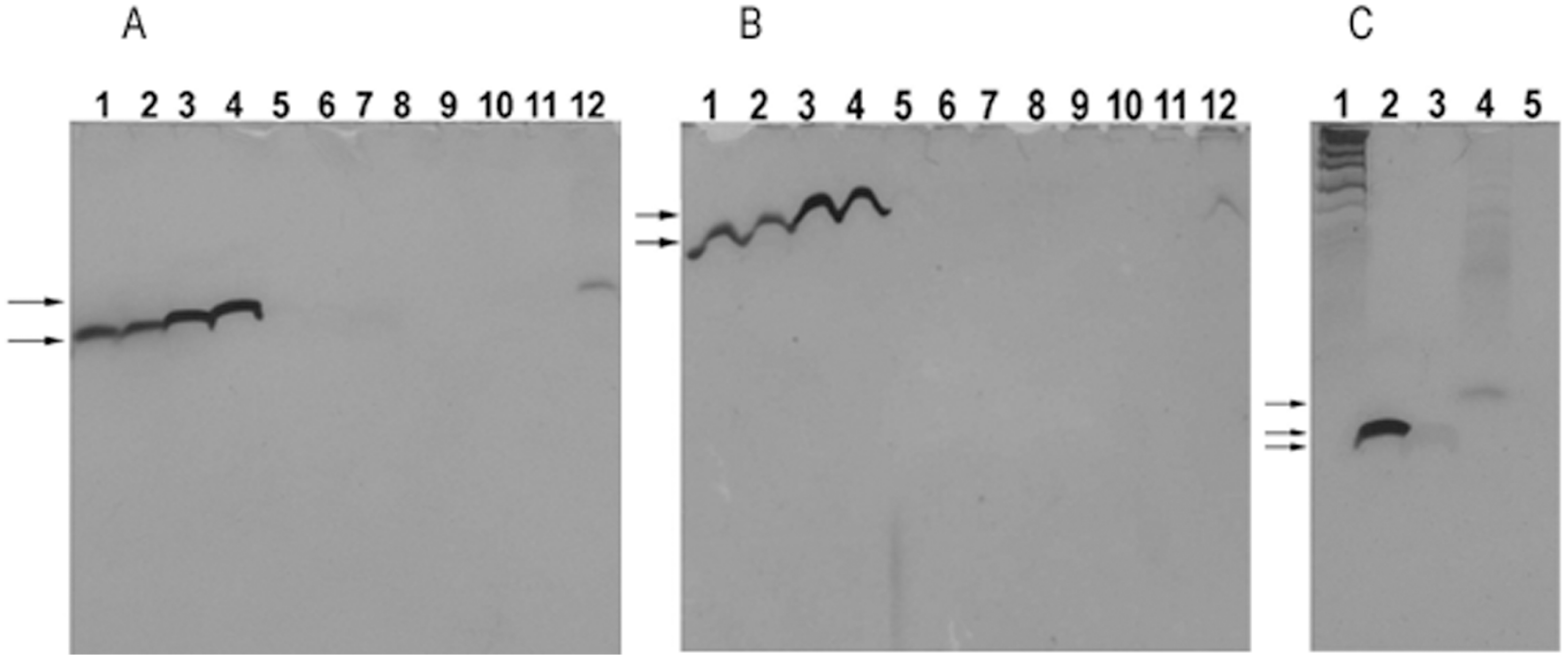
5-8-nt long oligos (dA), (dC), (dG) and (dT) purified by HPLC were silver-stained in denaturing PAGE gels. DNA markers were DNA marker 1 and oligo A_8_. The arrows indicate the specific oligo bands. (A) and (B) 25% and 45% gels. Lanes 1–12: oligos A_5_, A_6_, A_7_, A_8_, C_5_, C_6_, C_7_, T_5_, T_6_, T_7_, T_8_ and G_5_. (C) 20% gel. Lanes 1–5: DNA marker 1, oligos A_8_, C_8_, G_5_ and T_8_. Note: We are sorry that some DNA bands shown are bad. We show these bad DNA bands because we think that the detection rather than the size of oligo bands is critical. We think that the bad DNA bands were caused by the following reasons: 1. It was not easy to get the nice oligo bands in a high-concentration gel, especially when the gel concentration was higher than 25% because a high voltage had to be applied to the high-concentration gel to move the oligos; 2. The gels were overstained or overexposed to show the weak oligo bands; 3. Some oligos were not pure enough to eliminate the nonspecific bands; 4. Single-strand oligos detected were easy to form secondary structure and generate nonspecific bands even in a denaturing gel, especially when the oligos were G-rich; 5. A short oligo could diffuse in a low-concentration PAGE gel and generate a bad oligo band or background; 6. Some DNA bands of DNA marker 1 (GeneRuler^™^ Ultra low Range DNA ladder, Fermentas, Lithuania) were hard to be silver-stained.

**Fig 3:**
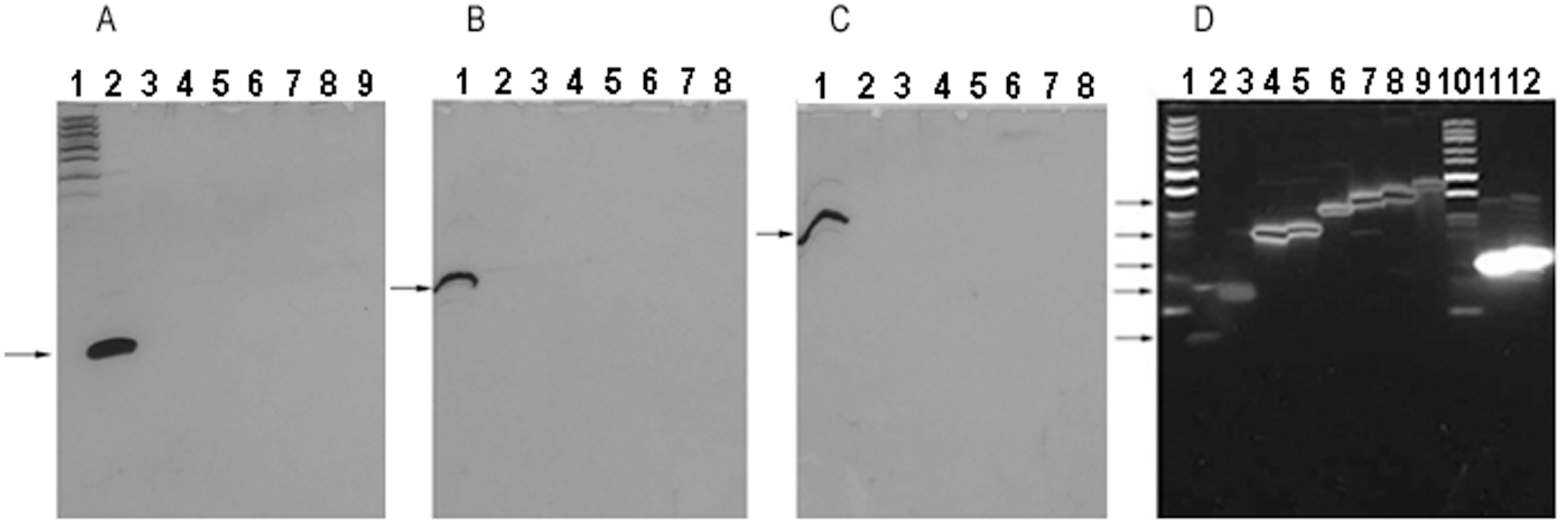
Oligos T_14_, T_29_, T_31_, T_40_, T_45_, T_50_, T_59_, A_12_T_12_ and (TA)_12_ in 15%, 30% and 40% denaturing PAGE gels. DNA markers were DNA marker 1 and oligo A_9_. The arrows indicate the specific oligo bands. (A), (B) and (C) 15%, 30% and 40% gels silver-stained. Lanes 1–9 of (A): DNA marker 1, oligos A_9_, T_14_, T_29_, T_31_, T_40_, T_45_, T_50_ and T_59_. Lanes 1–8 of (B) and (C): Oligos A_9_, T_14_, T_29_, T_31_, T_40_, T_45_, T_50_ and T_59_. (D), 15% gel stained with GDD. Lanes 1–12: DNA marker 1, oligos A_9_, T_14_, T_29_, T_31_, T_40_, T_45_, T_50_, T_59_, A_12_T_12_, and (TA)_12_. Note: We are sorry that some DNA bands shown are bad. The reasons why these DNA bands are bad have been mentioned in the figure legend of Fig 2.

2. Generally, the absolute values of Δ IntDen of most oligos would increase as the gel concentration increased. The shorter the oligo was, the more difficult its silver staining was. Based on the absolute values of the average Δ IntDen, the sensitivity of oligos (dA), (dC), (dG) and (dT) to silver staining could be ranged as (dA) > (dG) > (dC) > (dT) from high to low when the oligos were ≤ 8 nt long. Oligos C_5_, C_6_ and 5-59-nt long oligo (dT) were hard to be silver-stained (Table 1, Figs 2 and 3). The absolute values of the average Δ IntDen of oligo-sets could be ranged as oligo-sets (G-A) > (A-G) > (A-C) > (C-A) > (T-A) > (G-C) > (A-T) > (C-G) > (T-G) > (G-T) > (T-C) > (C-T) from high to low. In oligo-sets (A-C), (C-A), (A-G) and (G-A), the absolute value of Δ IntDen usually increased as base A increased, but the absolute value of Δ IntDen of oligo-set (G-A) ceased to increase or even decrease when base A was > 3 nt. In oligo-sets (C-G) and (G-C), the absolute value of Δ IntDen usually increased as base G increased.

3. The silver staining of an oligo containing base T could be partially or even completely inhibited by base T. For example, oligos A_3_T_5_, A_4_T_4_, G_5_T_3_, and G_4_T_4_ could be stained with SGRGS in 20% gel, but could not be silver-stained, meaning that the silver staining of these oligos had been completely inhibited by base T in 20% gel. The absolute values of Δ IntDen of oligos A_5_T_3_, C_7_T, and G_5_T_3_ were lower than those of oligos A_5_, C_7_, and G_5_, respectively, meaning that the silver staining of these oligos had been partially inhibited by base T (Table 1). Here bases A, C and G are called as the argyrophil nucleobases, while base T is called as the anti-silver nucleobase. Generally, the absolute value of Δ IntDen of oligos silver-stained would increase with the increase of the argyrophil nucleobases, or with the decrease of base T.

### Cyanine staining

**Fig 4:**
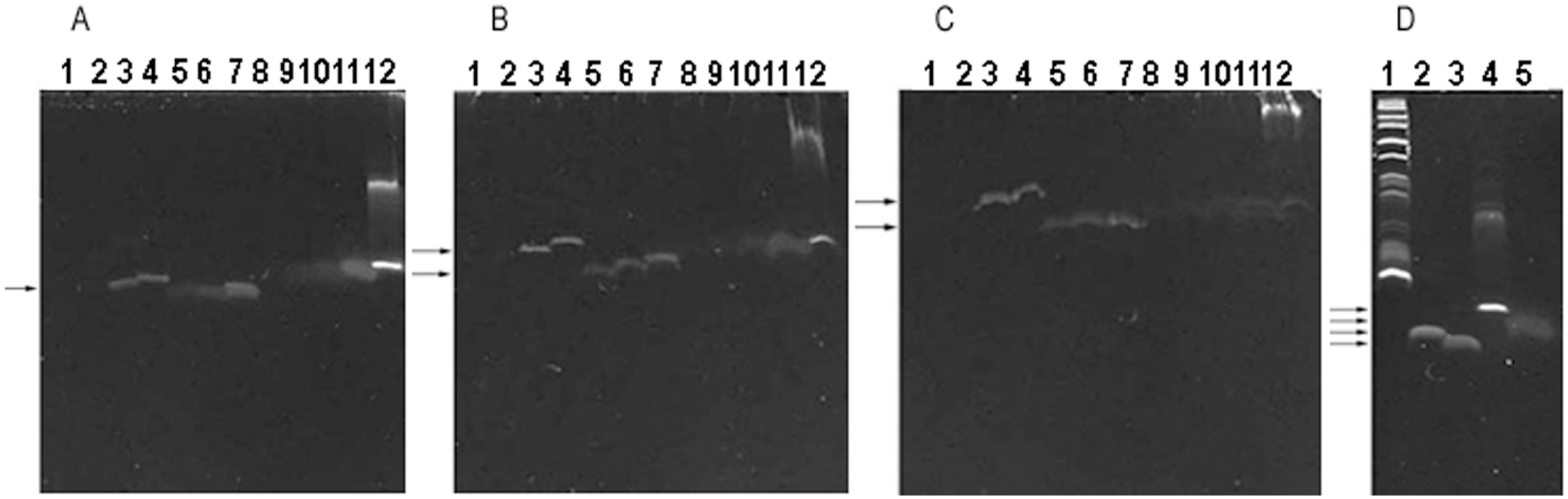
5-8-nt long oligos (dA), (dC), (dG) and (dT) purified by HPLC were stained with GDD in denaturing PAGE gels. DNA markers were DNA marker 1 and oligo A_8_. The arrows indicate the specific oligo bands. (A), (B) and (C) 20%, 30% and 40% gels. Lanes 1–12: Oligos A_5_, A_6_, A_7_, A_8_, C_5_, C_6_, C_7_, T_5_, T_6_, T_7_, T_8_ and G_5_. (D) 20% gel. Lanes 1–5: DNA marker 1, oligos A_8_, C_8_, G_5_ and T_8_. Note: We are sorry that some DNA bands shown are bad. The reasons why these DNA bands are bad have been mentioned in the figure legend of Fig 2.

1. S1A2–S1K2 Fig show the oligo bands in 35% gel stained with SGRGS. The bands of oligo (dT) were wide or even dispersive comparing with the bands of oligos (dA), (dC) and (dG), especially, in the low-concentration gels (Fig 4, S1H2 and S1I2 Fig and S2K2–S2O2 Fig). The oligo bands of 0.01–1μg of oligo A_11_ stained with asymmetric cyanine were shown in Fig 1B and 1C. GDD staining of some oligos was equivalent to or even better than SGRGS staining (Fig 1B and 1C and S3 Fig). Additionally, if the gel was not fixed with methanol fixing solution before staining, oligo A_11_ was difficult to be stained with GDD even if its loading amount was increased up to 10 μg per well (Fig 1D).

2. Δ IntDen of all the oligos stained with cyanine was also listed in Table 1. Generally, the Δ IntDen of most oligos would increase as the gel concentration increased. Additionally, the shorter an oligo was, the more difficult its cyanine staining was. If an oligo could be stained with SGRGS or GDD, its band should be brighter than the background. Therefore the Δ IntDen of an oligo band stained with SGRGS or GDD should be a positive number. Δ IntDen (cyanine staining) in Table 1 changes from 0 to 562867. The oligo band would be pretty weak if its Δ IntDen was < 3,500. The Δ IntDen of 0.01–1μg of oligo A_11_ (cyanine staining) was also listed in S2 Table. The Δ IntDen from those hazy oligo-bands was also recorded in italics in Table 1.

3. According to the average Δ IntDen, the sensitivity of oligos (dA), (dC), (dG) and (dT) to GDD staining could be ranged as (dG) > (dC) > (dT) > (dA) from high to low when the oligos were ≤ 7 nt long. For example, oligos C_5_, T_5_ and G_5_ could be stained with GDD in 15–45% gels, but oligos A_5_ and A_6_ were hard to be stained (Table 1 and Fig 4). The average Δ IntDen of oligo (dA) was higher than that of oligo (dC) when they were 8 nt long. Therefore the sensitivity of oligos (dA), (dC) and (dT) to GDD staining could be changed into (dA) > (dC) > (dT) from high to low when the oligos were 8 nt long. Since a > 5-nt long oligo (dG) was not available for us, we could not determine the Δ IntDen of a > 5-nt long oligo (dG). When oligo-sets were 8 nt long, the average Δ IntDen of different oligo-sets could be ranged as oligo-sets (T-G) > (G-T) > (A-G) > (G-A) > (C-G) > (T-A) > (G-C) > (A-T) > (C-A) > (T-C) > (C-T) > (A-C) from high to low.

4. In oligo-set (A-C), the Δ IntDen of oligos in 35–40% gel would obviously increase when base C accumulated to > 5 nt. Moreover, the Δ IntDen of oligo AC_7_ located at C-side were higher than that of oligo A_4_C_4_ located in the middle or that of oligo A_7_C located at A-side. In the oligo-sets containing base G such as oligo-sets (A-G) and (T-G), the Δ IntDen increased with the increase of base G. Moreover, the Δ IntDen of oligo-sets (A-G), (G-A) and (G-C) would suddenly and significantly increased when base G accumulated to ≥ 4 nt, for example, the Δ IntDen of oligos A_5_G_3_, A_6_G_2_, and A_7_G in 15% gel were 5815, 0, and *640*, respectively, whereas the Δ IntDen of oligo A_4_G_4_ was 53666, about 9.2 folds higher than that of oligo A_5_G_3_. The Δ IntDen of oligo (AG_3_)_2_ was even about 56.5 folds higher than that of oligo A_5_G_3_. In oligo-sets (A-T), (T-A), (C-T) and (T-C), the Δ IntDen increased with the increase of base T. Moreover, the Δ IntDen of oligo-set (A-T) and (T-A) would increase obviously when base T accumulated to ≥ 4 nt (Table 1). In oligo-sets (C-A), (C-T) and (T-C), the Δ IntDen of the oligos located at 2 sides of these oligo-sets was higher than that of the oligos located in the middle. But in oligo-set (C-G), the closer to the middle of the oligo-set, the higher the Δ IntDen was.

5. The Δ IntDen of an oligo located in the middle of oligo-set (A-C), (C-A), (C-T) or (T-C) was lower than the total Δ IntDen calculated according to each nucleobase in the oligo. For example, the Δ IntDen of oligos A_7_ and C_7_ in 35% gels were 3961 and 11204, respectively. Thus, the Δ IntDen of each base A or C should be 565.86 (3961/7) or 1600.57 (11204/7), respectively. Therefore the total Δ IntDen of 4 nt of base A and 4 nt of base C should be 8665.72 (4 × 565.86 + 4 × 1600.57), much higher than the actual Δ IntDen of oligo A_4_C_4_ or C_4_A_4_ (Table 1). Although the average Δ IntDen of oligo (dA) was lower than that of oligo (dC) when they were < 8 nt long, the average Δ IntDen of oligo-set (A-G) or (A-T) was higher than that of oligo-set (C-G) or (C-T), respectively. These findings mean that the Δ IntDen of an oligo is not always equivalent to the total Δ IntDen calculated according to each nucleobase in the oligo.

6. The Δ IntDen of oligo GA_7_ or CA_7_ was obviously higher than that of oligo A_7_G or A_7_C, respectively, indicating that SGRGS staining of some oligos could be affected by the base-sequence direction. The Δ IntDen of oligo (dA), (dC), (dG) and (dT) could be changed when a base A, C, G or T was added to their 5′- or 3′-end. For example, when a base C, G, or T was added to the 5′-end of oligo A_7_ to form oligo CA_7_, GA_7_, or TA_7_, the Δ IntDen of oligos CA_7_, GA_7_, and TA_7_ was usually higher than that of oligo A_7_, but lower when a base C or T was added to its 3′-end (Table 1). There are some oligo isomers in Table 1, such as isomers A_3_G_5_/A_2_G_3_AG_2_, (CG_3_)_2_/CGCG_5_, G_3_AG_4_/G_5_AG_2_, and G_5_TG_2_/G_2_TG_5_ which have the same base composition, but have different base sequences. The Δ IntDen of some oligo isomers was not always equivalent. For example, Δ IntDen of isomers G_3_AG_4_/G_5_AG_2_ was equivalent in silver staining, but obviously different in SGRGS staining.

7. The migration rate of oligos A_8_, C_8_, G_5_, and T_8_ in the denaturing PAGE gel could be ranged as oligos C_8_ >A_8_ >T_8_ > G_5_ from high to low (Fig 4 and S1H2 and S1I2 Fig). According to the findings in S1 and S4 Figs, the migration rate of oligos A_4_C_4_, A_4_G_4_, A_4_T_4_, C_4_A_4_, C_4_G_4_, C_4_T_4_, G_4_A_4_, G_4_C_4_, G_4_T_4_, T_4_A_4_, T_4_C_4_ and T_4_G_4_ could be ranged as oligo C_4_T_4_ = T_4_C_4_ ≥ A_4_C_4_ = C_4_A_4_ > A_4_T_4_ = T_4_A_4_ >A_4_G_4_ = G_4_A_4_ = G_4_T_4_ = T_4_G_4_ > C_4_G_4_ = G_4_C_4_ from high to low. The molecular weights of these oligos could be ranged as A_4_G_4_ = G_4_A_4_ (1144.8) > G_4_T_4_ = T_4_G_4_ (1108.8) > C_4_G_4_ = G_4_C_4_ (1048.8) > A_4_T_4_ = T_4_A_4_ (1044.8) > A_4_C_4_ = C_4_A_4_ (984.8) > C_4_T_4_ = T_4_C_4_ (948.8) from high to low. Therefore the migration rate of oligos was not always consistent with their molecular weights even in the denaturing PAGE gels. Base G was the major nucleobase decreasing the migration rate.

## Discussion

Most of the oligos could be stained with silver or cyanin in our experiments. Both silver staining and cyanine staining could complement each other, for example, oligo (dT) was able to be stained with cyanine, but hard to be silver-stained. Oligo A_5_ was able to be silver-stained, but hard to be stained with cyanine. One exception was that both of them were insensitive to the oligo-sets (C-T) and (T-C) (Table 1 and S2 Fig).

The mechanism of DNA silver-staining is that Ag^+^ binds to nucleobases, and then is selectively reduced to Ag° with chemical agents or light [12–15]. However, it is unclear why oligo (dT) is hard to be silver-stained and why the silver staining of an oligo containing base T can be inhibited by base T. Shukla and Sastry have reported that the binding affinities of nucleobases for silver ions go C > G > A ≥ T in order [21], which seem able to explain why oligo (dT) is hard to be silver-stained. However, it can not explain why the silver-staining of the oligos containing base T could be inhibited by base T and why the sensitivity of oligos to silver staining is (dA) > (dG) > (dC) > (dT) in order from high to low. Base T has two keto groups: one is at position 2 of purine ring, and another is at position 4 of purine ring. Base C or G has only one keto group at position 2 of pyrimidine ring or position 6 of purine ring, respectively. Base A lacks keto group. Therefore we speculate that perhaps the oxygen atoms in the two keto groups of base T can capture the silver cation, and hence interfere or interrupt the reduction from silver cation to metallic silver. One oxygen atom in the keto group of base C or base G might not be enough to interrupt the silver staining.

**Fig 5:**
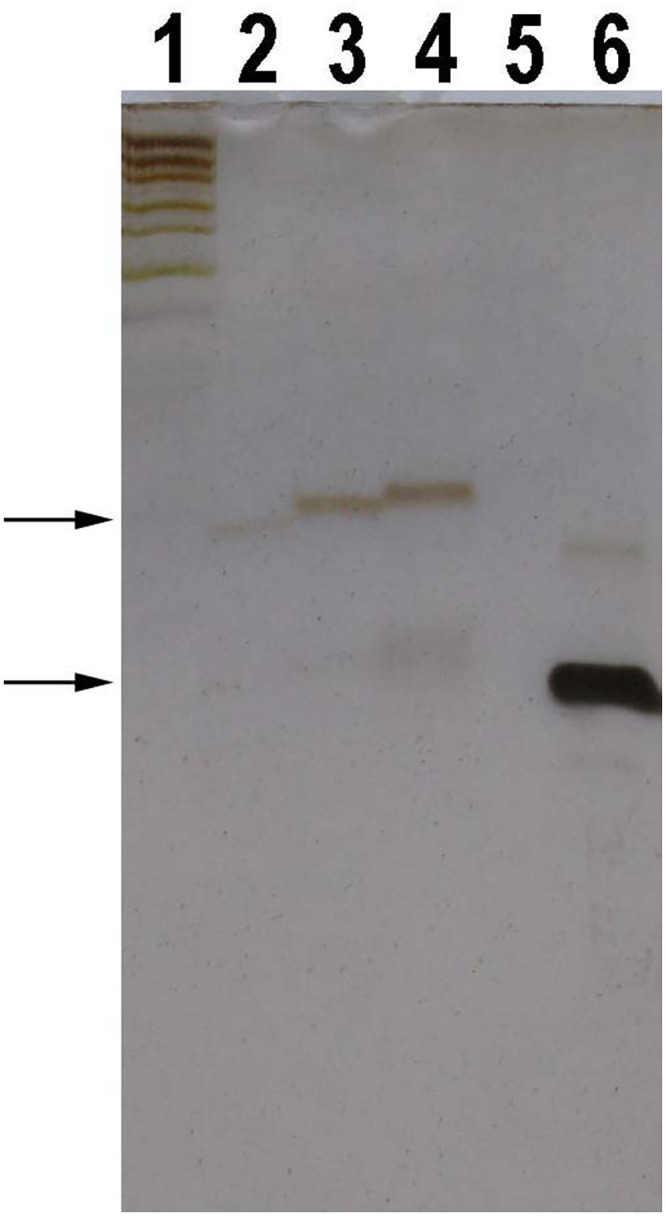
Oligos A_10_T_10_, A_11_T_11_, A_12_T_12_ and (TA)_12_ were silver-stained in 15% denaturing gel. DNA markers were DNA marker 1 and oligo A_9_. The arrows indicate the specific oligo bands. Lanes 1–6: DNA marker 1, oligos A_10_T_10_, A_11_T_11_, A_12_T_12_, (TA)_12_ and A_9_. Note: We are sorry that some DNA bands of DNA marker 1 are not clear because they are hard to be silver-stained.

The inhibition of silver staining by base T could be affected by the amounts of the argyrophil nucleobases and base T, the cis-distance between the argyrophil nucleobase and base T, and the gel concentration. Generally, the more base T was, or the less the argyrophil nucleobases were, or the shorter the cis-distance was, the stronger the inhibition of silver staining by base T was. For example, oligos A_5_T_5_, A_7_T_3_ and A_7_T_20_ could not be silver-stained in 15% gel, but oligos A_8_T_2_, A_10_T_10_, A_15_T_15_, and A_20_T_20_ could, indicating that ≤ 7 nt of base A were not enough to relieve the inhibition from ≥ 3 nt of base T in 15% gel, but 8, 10, 15 and 20 nt of base A were enough to relieve the inhibition from 2, 10, 15, and 20 nt of base T, respectively. Both oligos A_12_T_12_ and (TA)_12_could be stained with GDD (Fig 3D), but oligo (TA)_12_ in which the cis-distance between the argyrophil nucleobase and base T was shorter than oligo A_12_T_12_ was more difficult to be silver-stained than oligo A_12_T_12_ (Fig 5).

The inhibition of silver staining by base T could be partially relieved by increasing the gel concentration. For example, oligo A_6_T_2_ could be silver-stained in 25–40% gel, but could not in 15–20% gel. We suppose that the gel between the argyrophil nucleobase and base T can reduce the exposure of argyrophil nucleobase to base T, and hence the increase of the gel concentration can partially relieved the inhibition of silver staining by base T. The possible biological significance of the ability for base T to inhibit silver staining is unclear. As we know, there are many AT-rich regions in DNA, such as TATA box and the matrix-associated region (MAR), etc. Additionally, some DNA binding domains such as zinc finger contain metal atoms. Our findings might indicate that base T could interact with metal ions, which could be affected by the surrounding conditions such as the concentration of surrounding media, etc.

The migration rate of oligos A_8_, C_8_, G_5_, and T_8_ in denaturing PAGE gels could be ranged as oligos C_8_ > A_8_ > T_8_ > G_5_ from high to low. In another experiment, we found that the migration rate of oligos A_9_, C_9_, G_4_AG_4_, and T_9_ in a non-denaturing PAGE gel could also be ranged as oligos C_9_ > A_9_ > T_9_ > G_4_AG_4_ from high to low (S5A Fig). The molecular weight of bases A, C, G and T can be ranged as G (151.1) > A (135.1) > T (126.1) > C (111.1) from high to low. Base C or G can form 3 hydrogen bonds, while base A or T can form only 2 hydrogen bonds. Oligo (dC) always migrated the fastest, perhaps suggesting that oligo (dC) maintained a compact secondary structure or did not link themselves to form a larger molecular group by hydrogen bonds in our denaturing or non-denaturing PAGE gels.

The molecular weight of oligo G_5_ is the lowest among oligos A_8_, C_8_, G_5_ and T_8_. It is difficult to be completely explained using the molecular weight of oligo G_5_ that oligo G_5_ migrated even more slowly than oligo A_8_ in denaturing PAGE gel. In other experiments, we found that the bands of oligos (AG_3_)_2_AG, A_2_G_3_AG_4_ and A_3_G_3_AG_3_ migrated much more slowly than expected in a denaturing gel if these oligos were not denatured at 95°C (S5B and S5C Fig). In a non-denaturing gel, oligos (AG_3_)_2_, A_2_G_3_AG_2_, A_3_G_5_ and A_4_G_4_ had the same performance as oligos (AG_3_)_2_AG, A_2_G_3_AG_4_ and A_3_G_3_AG_3_ (S5D and S5E Fig). These findings indicated that the chains of oligo (dG) might tend to form a loose secondary structure or link themselves to form a larger molecular group by hydrogen bonds.

It was surprising that oligo T_8_ migrated even more slowly than oligo A_8_ because the molecular weight of base T was less than that of base A and could form only 2 hydrogen bonds as many as base A could. Some atoms in a nucleobase can affect the width of the cross sections of the nucleobase. These atoms in bases A, C, G and T are H-C-N-C-N-C-H, O-C-N-C-H, H-N-C-N-C-N-C-H, and O-C-N-C-C-H, respectively (S6 Fig). Their total atomic weights are 66, 55, 80 and 67, respectively. Therefore we suppose that the higher the total atomic weight is, the lower the migration rate is.

An oligo can diffuse from PAGE gel into the surrounding solution such as staining or washing solution. The shorter the oligos are, or the lower the gel concentration is, the faster the diffusion of the oligos is. The fixing of PAGE gels with methanol fixing solution can effectively prevent the diffusion of oligos. Oligo A_11_(0.04μg per well) could be stained with GDD in 15% gel if the gel was fixed, but could not even if its loading amount was increased up to 10 μg per well if the gel was not fixed, meaning that the sensitivity of GDD staining of oligo A_11_ could be enhanced about 250-folds (10/0.04) by fixing the gel with methanol fixing solution. It has to be mentioned that the fixing of a gel with methanol fixing solution could not completely stop the diffusion of oligos. We recommend that the gel-fixing time is 1.5 - 2hrs. If the gel-fixing time is too long, for example, overnight, there will be a risk that the silver or cyanine staining of oligos becomes weak or even fail. Low temperature can reduce the diffusion of short oligos and enhance the silver staining of short oligos [12]. Therefore we fixed, washed and stained the gels at 4°C when the oligos were shorter than 8 nt.

## Conclusion

The sensitivity of oligos (dA), (dC), (dG) and (dT) to silver staining could be ranged as (dA) > (dG) > (dC) > (dT) from high to low. Oligo (dT) was hard to be silver-stained. The silver staining of an oligo containing base T could be partially or completely inhibited by base T. The inhibition of silver staining by base T could be affected by the amounts of the argyrophil nucleobase and base T, the cis-distance between the argyrophil nucleobase and base T, and the gel concentration; 2. The sensitivity of oligos (dA), (dC), (dG) and (dT) to GDD staining could be ranged as (dG) > (dC) > (dT) > (dA) from high to low when they were ≤ 7 nt long, or (dA) > (dC) > (dT) when they were 8 nt long; 3. Silver staining and cyanine staining could complement each other, but they were insensitive to oligo-sets (C-T) and (T-C); 4. The migration rate of oligos A_8_, C_8_, G_5_, and T_8_ in denaturing PAGE gel could be ranged as oligos C_8_ > A_8_ > T_8_ > G_5_ from high to low. Base G was the major nucleobase decreasing the migration rate.

## Supporting Information

S1 Fig The oligo bands of 8-nt long oligo-sets in 35% denaturing PAGE gels stained with silver and SGRGS. (PDF)

S2 Fig Oligos A_8_, C_7_, C_8_, G_5_, T_6_, T_7_, T_8_ and Oligo-sets (C-T), (T-C), (G-T) and (T-G) in 15–40% denaturing PAGE gels stained with silver and cyanine. (PDF)

S3 Fig Oligo-sets (A-C), (C-A) and (G-C) in denaturing PAGE gels stained with GDD and SGRGS. (PDF)

S4 Fig Oligos A_4_C_4_, A_4_G_4_, A_4_T_4_, C_4_A_4_, C_4_G_4_, C_4_T_4_, G_3_AGA_3_, G_4_C_4_, G_4_T_4_, T_4_A_4_, T_4_C_4_ and T_4_G_4_ in 30% denaturing PAGE gel. (PDF)

S5 Fig Oligos T_9_, G_4_AG_4_, C_9_, A_9_ and oligo-set (A-G) in different PAGE gel. (PDF)

S6 Fig A schematic diagram of the atoms which can affect the width of the cross sections of bases A, C, G and T that are vertical to the glycosidic bond between the base and the deoxyribose. (PDF)

S1 Table The sequences of synthesized oligos. (PDF)

S2 Table Δ IntDen of oligo A_11_ stained with Silver and SGRGS. (PDF)
