# Supplementary material for: Silver and Cyanine Staining of Oligonucleotides in Polyacrylamide Gel
Source: PLoS One. 2015 Dec 9;10(12):e0144422. doi: 10.1371/journal.pone.0144422 (PMC4674134; doi:10.1371/journal.pone.0144422)
Supplement: S1 Fig — (PDF) [file pone.0144422.s001.pdf]

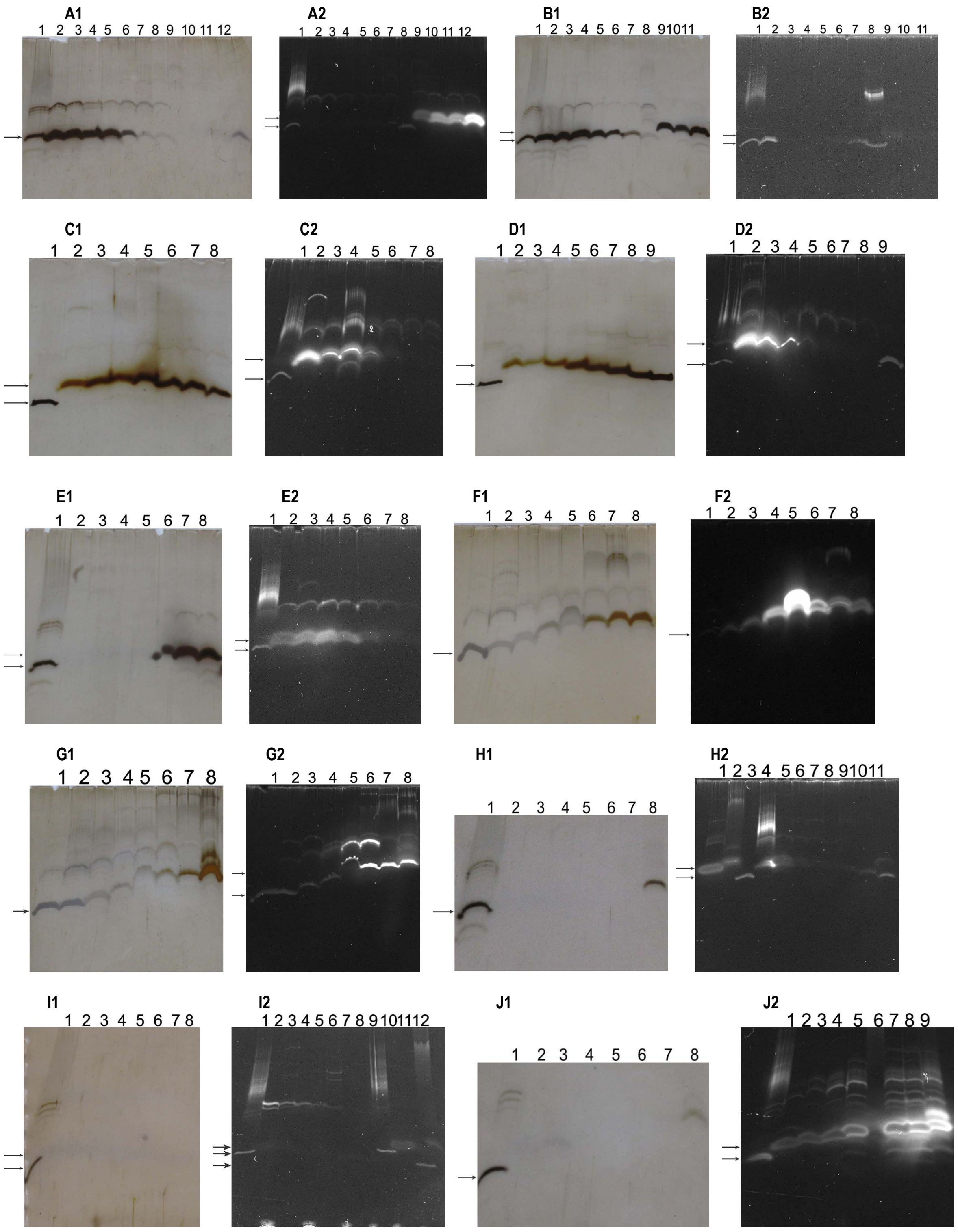
A1
1 2 3 4 5 6 7 8 9 10 11 12
A2
1 2 3 4 5 6 7 8 9 10 11 12
B1
1 2 3 4 5 6 7 8 9 10 11
B2
1 2 3 4 5 6 7 8 9 10 11
C1
1 2 3 4 5 6 7 8
C2
1 2 3 4 5 6 7 8
D1
1 2 3 4 5 6 7 8 9
D2
1 2 3 4 5 6 7 8 9
E1
1 2 3 4 5 6 7 8
E2
1 2 3 4 5 6 7 8
F1
1 2 3 4 5 6 7 8
F2
1 2 3 4 5 6 7 8
G1
1 2 3 4 5 6 7 8
G2
1 2 3 4 5 6 7 8
H1
1 2 3 4 5 6 7 8
H2
1 2 3 4 5 6 7 8 9 10 11
I1
1 2 3 4 5 6 7 8
I2
1 2 3 4 5 6 7 8 9 10 11 12
J1
1 2 3 4 5 6 7 8
J2
1 2 3 4 5 6 7 8 9

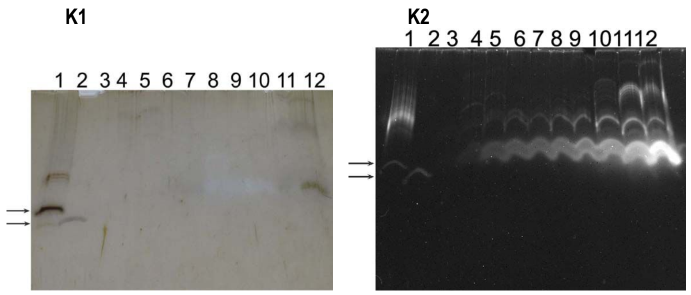


**S1 Fig. The oligo bands of 8-nt long oligo-sets in 35% denaturing PAGE gels stained with silver and SGRGS.** The oligos were purified by using HAP after syntheses. DNA markers were oligos $A_8$ and $C_9$. The arrows indicate the specific oligo bands. (A1)-(K1) were silver-stained. (A2)-(K2) were stained with SGRGS. (A1) and (A2) Oligo-sets (A-C) and (A-T). Lanes 1-12: Oligos $A_8$, $A_7C$, $A_6C_2$, $A_5C_3$, $A_4C_4$, $A_3C_5$, $A_2C_6$, $AC_7$, $AT_7$, $A_2T_6$, $A_3T_5$ and $A_4T_4$. (B1) and (B2) Oligo-sets (C-A) and (A-T). Lanes 1-11: oligos $A_8$, $CA_7$, $C_2A_6$, $C_3A_5$, $C_4A_4$, $C_5A_3$, $C_6A_2$, $C_7A$, $A_5T_3$, $A_6T_2$ and $A_7T$. (C1) and (C2) Oligo-set (A-G). Lanes 1-8: Oligos $A_8$, $(AG_3)_2$, $A_2G_3AG_2$, $A_3G_5$, $A_4G_4$, $A_5G_3$, $A_6G_2$ and $A_7G$. (D1) and (D2) Oligo-set (G-A). Lanes 1-9: Oligos $A_8$, $G_5AG_2$, $G_3AG_4$, $(G_3A)_2$, $G_3AG_2A_2$, $G_3AGA_3$, $G_3A_5$, $G_2A_6$ and $GA_7$. (E1) and (E2) Oligo-set (T-A). Lanes 1-8: Oligos $A_8$, $T_7A$, $T_6A_2$, $T_5A_3$, $T_4A_4$, $T_3A_5$, $T_2A_6$ and $TA_7$. (F1) and (F2) Oligo-set (C-G). Lanes 1-8: Oligos $C_9$, $C_7G$, $C_6G_2$, $C_5G_3$, $C_4G_4$, $C_3G_5$, $CGCG_5$ and $(CG_3)_2$. (G1) and (G2) Oligo-set (G-C). Lanes 1-8: Oligos $C_9$, $GC_7$, $G_2C_6$, $G_3C_5$, $G_4C_4$, $G_5C_3$, $G_5C_2G$ and $G_5CG_2$. (H1) Oligo-set (C-T). Lanes 1-8: Oligos $A_8$, $CT_7$, $C_2T_6$, $C_3T_5$, $C_4T_4$, $C_5T_3$, $C_6T_2$ and $C_7T$. (H2) Oligo-set (C-T) and oligos $A_8$, $C_8$, $G_5$ and $T_8$. Lanes 1-11: Oligos $T_8$, $G_5$, $C_8$, $A_8$, $CT_7$, $C_2T_6$, $C_3T_5$, $C_4T_4$, $C_5T_3$, $C_6T_2$ and $C_7T$. (I1) Oligo-set (T-C). Lanes 1-8: Oligos $A_8$, $T_7C$, $T_6C_2$, $T_5C_3$, $T_4C_4$, $T_3C_5$, $T_2C_6$ and $TC_7$. (I2) Oligo-set (T-C) and oligos $A_8$, $C_8$, $G_5$ and $T_8$. Lanes 1-12: Oligos $A_8$, $T_7C$, $T_6C_2$, $T_5C_3$, $T_4C_4$, $T_3C_5$, $T_2C_6$, $TC_7$, $A_8$, $T_8$, $C_8$ and $G_5$. (J1) Oligo-set (G-T). Lanes 1-8: Oligos $A_8$, $GT_7$, $G_2T_6$, $G_3T_5$, $G_4T_4$, $G_5T_3$, $G_5TGT$, and $G_5TG_2$. (J2) Oligo-set (G-T). Lanes 1-9: Oligos $A_8$, $GT_7$, $G_2T_6$, $G_3T_5$, $G_4T_4$, $G_5$,

$G_5T_3$, $G_5TGT$, and $G_5TG_2$. (K1) and (K2) Oligo-set (T-G). Lanes 1-12: Oligos $A_8$, $C_8$, $T_6$, $T_7$, $T_8$, $T_7G$, $T_6G_2$, $T_5G_3$, $T_4G_4$, $T_3G_5$, $TGTG_5$ and $G_2TG_5$.

In oligo-sets (A-C), (C-A), (A-G) and (G-A), all the oligos could be silver-stained and the oligo bands became stronger and stronger as base A increased, but cease to increase obviously when base A was ≥ 4 nt (A1-D1). The bands of oligos $A_7C$, $A_6C_2$, $A_5C_3$, $A_4C_4$, $A_3C_5$, $C_2A_6$, $C_3A_5$ and $C_4A_4$ stained with SGRGS were hardly visible or invisible (A2 and B2). The oligo bands of oligo-sets (A-G) and (G-A) stained with SGRGS became stronger and stronger as base G increased (C2 and D2). Oligos $AT_7$, $A_2T_6$, $A_3T_5$, $T_7A$, $T_6A_2$, $T_5A_3$ and $T_4A_4$ could not be silver-stained (A1, B1 and E1). The oligo bands of oligo-sets (A-T) and (T-A) stained with SGRGS were visible and became stronger and stronger as base T increased, but cease to increase obviously when base T was ≥ 4 nt (A2, B2 and E2). In oligo-sets (A-G), (G-A), (C-G) and (G-C), the migration rate of oligos decreased gradually with the increase of base G, but cease to decrease when base G was ≥ 4 nt. Oligos $C_4G_4$ and $G_4C_4$ migrated the most slowly (C1, D1, F1 and G1). The oligo bands of oligo-sets (C-G) and (G-C) silver-stained were grey when base C was ≥ 4 nt, or brown when base G was > 4 nt, indicating that oligo base composition could affect not only the intensity of an oligo band but also its color. In oligo-set (C-G), all the oligos could be stained with SGRGS. The closer to the middle of the oligo-set, the stronger the oligo bands were. The band of oligo $C_4G_4$ was the strongest (F2). In oligo-set (G-C), all the oligos could be stained with SGRGS and the oligo bands became much stronger when base G was ≥ 4 nt than when base G was < 4 nt (G2). Oligo-set (C-T) could not be silver-stained. The band in the lane of oligo $C_7T$ was a nonspecific band (H1). The oligo bands of oligo-set (T-C) were hazy or invisible (I1). The oligo bands of oligo-set (C-T) and (T-C) stained with SGRGS were weak, but the oligo bands of oligo-set (C-T) were stronger than

those of oligo-set (T-C). Additionally, the oligo bands of oligo-sets (C-T) and (T-C) migrated more and more slowly as base T increased (H2 and I2). In oligo-set (G-T), only the band of oligo $G_5TG_2$ silver-stained was clear, the other bands were weak, hazy or invisible (J1). The oligo bands stained with SGRGS became stronger and stronger and migrated more and more slowly as base G increased (J2). In oligo-set (T-G), the oligo bands silver-stained were hazy or invisible (K1). All the oligos could be stained with SGRGS. The oligo bands became stronger and stronger as base G increase, but the migration rate did not obviously change when base G increased (K2). There was a central pale-staining area in the most of oligo bands of oligo-sets (A-T), (T-A), (G-T) and (T-G) stained with SGRGS, especially when base T was ≥ 2 nt (A2, B2, E2, J2 and K2). Note: We are sorry that some DNA bands shown are bad. The reasons why these DNA bands are bad have been mentioned in the figure legend of Fig 2.
