# Supplementary material for: Silver and Cyanine Staining of Oligonucleotides in Polyacrylamide Gel
Source: PLoS One. 2015 Dec 9;10(12):e0144422. doi: 10.1371/journal.pone.0144422 (PMC4674134; doi:10.1371/journal.pone.0144422)
Supplement: S2 Fig — (PDF) [file pone.0144422.s002.pdf]

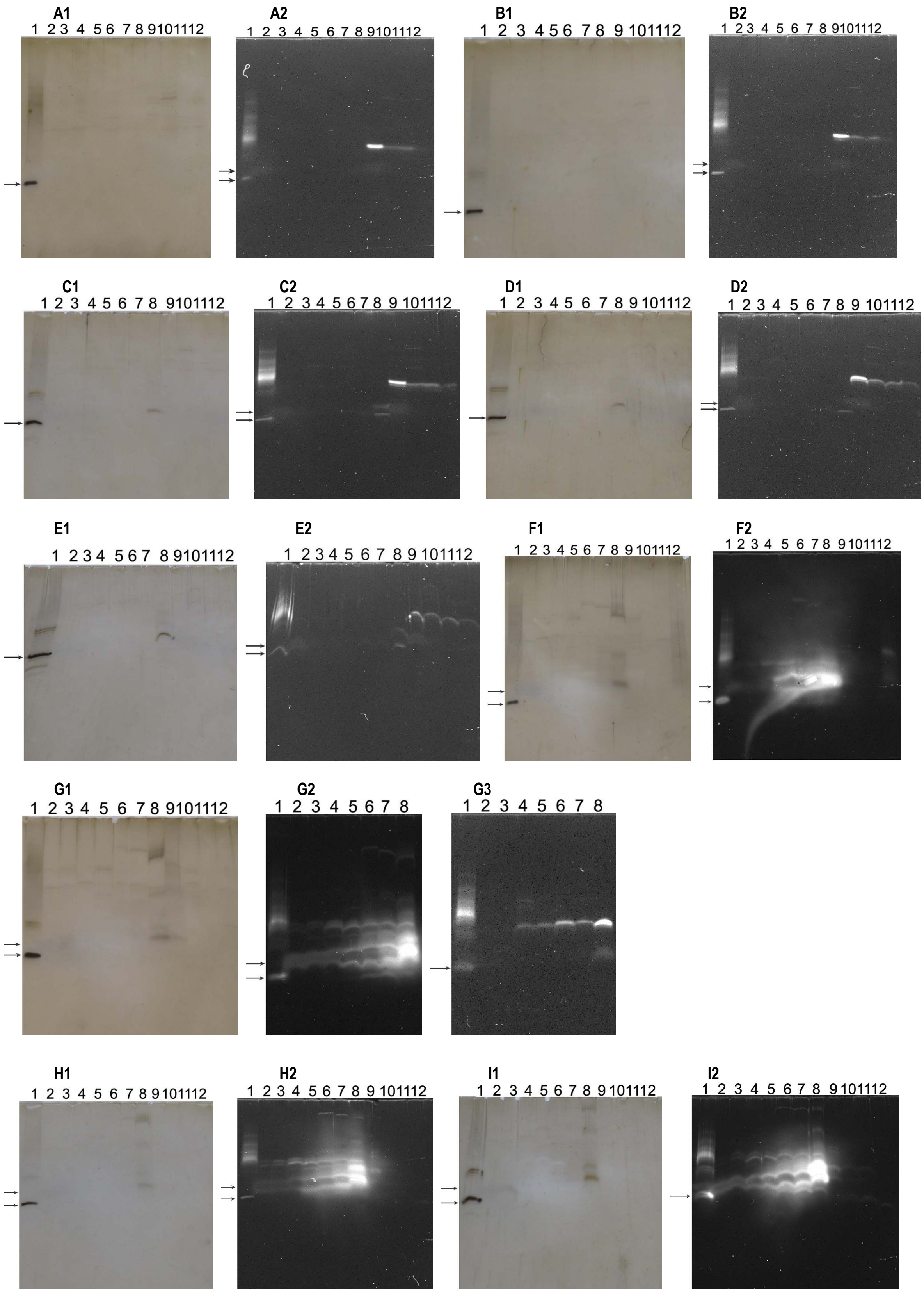
A1
1 2 3 4 5 6 7 8 9 10 11 12
A2
1 2 3 4 5 6 7 8 9 10 11 12
B1
1 2 3 4 5 6 7 8 9 10 11 12
B2
1 2 3 4 5 6 7 8 9 10 11 12
C1
1 2 3 4 5 6 7 8 9 10 11 12
C2
1 2 3 4 5 6 7 8 9 10 11 12
D1
1 2 3 4 5 6 7 8 9 10 11 12
D2
1 2 3 4 5 6 7 8 9 10 11 12
E1
1 2 3 4 5 6 7 8 9 10 11 12
E2
1 2 3 4 5 6 7 8 9 10 11 12
F1
1 2 3 4 5 6 7 8 9 10 11 12
F2
1 2 3 4 5 6 7 8 9 10 11 12
G1
1 2 3 4 5 6 7 8 9 10 11 12
G2
1 2 3 4 5 6 7 8
G3
1 2 3 4 5 6 7 8
H1
1 2 3 4 5 6 7 8 9 10 11 12
H2
1 2 3 4 5 6 7 8 9 10 11 12
I1
1 2 3 4 5 6 7 8 9 10 11 12
I2
1 2 3 4 5 6 7 8 9 10 11 12

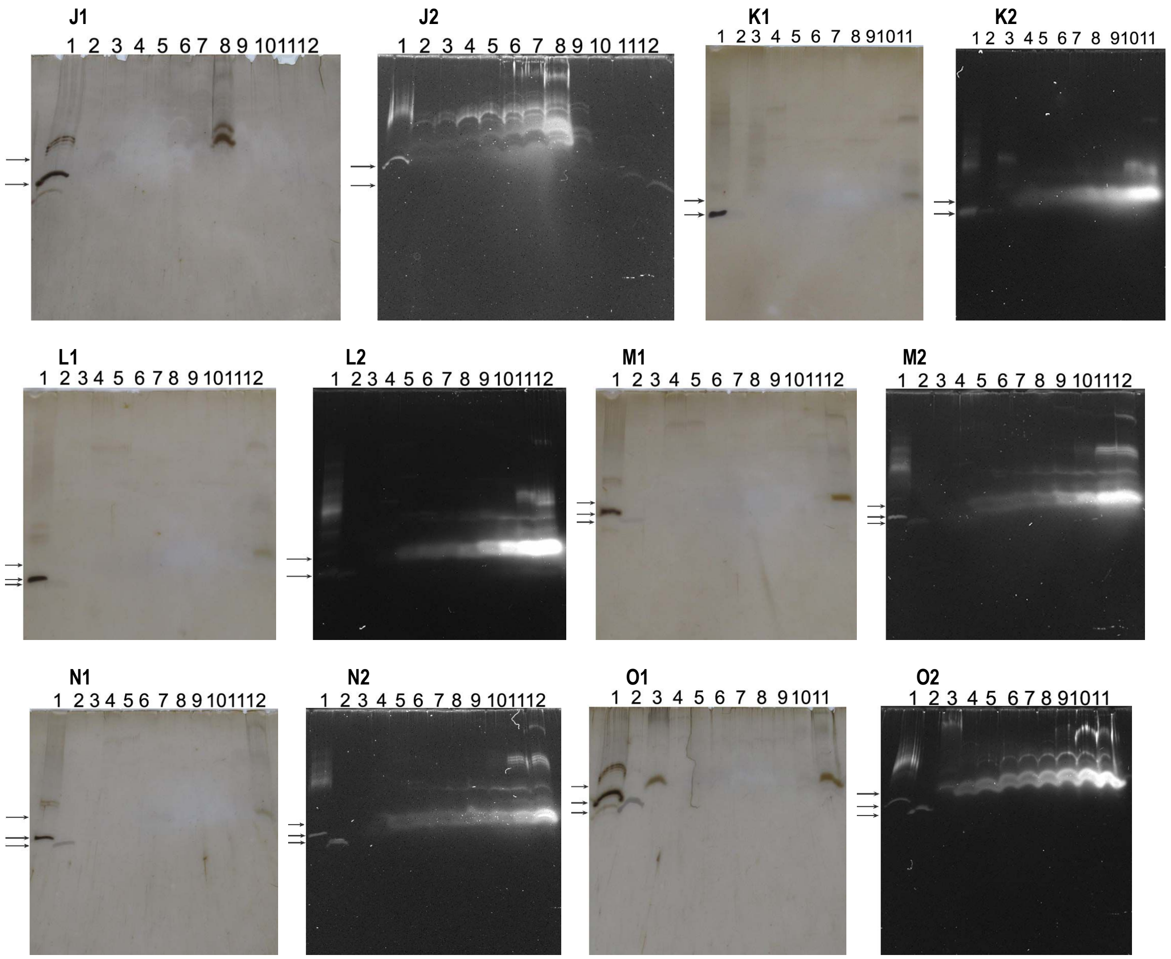

S2 Fig. Oligos A_8, C_7, C_8, G_5, T_6, T_7, T_8 and Oligo-sets (C-T), (T-C), (G-T) and (T-G) in 15-40% denaturing PAGE gels stained with silver and cyanine. DNA marker was oligo A_8. The arrows indicate the specific oligo bands. (A1)-(O1) were silver-stained. (A2)-(O2) and (G3) were stained with SGRGS. (A), (B), (C), (D) and (E) Oligo-sets (C-T) and (T-C) in 15%, 20%, 25%, 30% and 40% denaturing gels, respectively. Lanes 1-12: Oligos A_8, CT_7, C_2T_6, C_3T_5, C_4T_4, C_5T_3, C_6T_2, C_7T, T_7C, T_6C_2, T_5C_3 and T_4C_4. In (A1)-(E1), the specific oligo bands except that of oligo A_8 were hazy or invisible. The band in lane 8 of (C1), (D1) or (E1) was a nonspecific band. In (A2), (B2), (C2) and (D2), the bands of oligos A_8, CT_7, C_7T and T_7C was easily visible. The other oligo bands were very weak or invisible. In (E2), all of the oligo bands were weak although they were visible. (F1) and (F2) Oligo-sets (G-T) and (T-C) in 15%

denaturing gels. Lanes 1-12: oligos A_8, GT_7, G_2T_6, G_3T_5, G_4T_4, G_5T_3, G_5TGT, G_5TG_2, T_3C_5, T_2C_6, TC_7 and C_7. There was a band in lane 8 of (F1). The other oligo bands of (F1) were hazy or invisible. The oligo bands of oligo-set (G-T) in (F2) were visible and became stronger and stronger with the increase of base G. The bands of oligos T_3C_5, T_2C_6, TC_7 and C_7 in (F2) were invisible. (G1) Oligo-sets (G-T) and (T-C) in 20% denaturing gels. Lanes 1-12: oligos A_8, GT_7, G_2T_6, G_3T_5, G_4T_4, G_5T_3, G_5TGT, G_5TG_2, G_5, T_3C_5, T_2C_6 and TC_7. There was a band in lane 8 or 9. The other oligo bands were hazy or invisible. (G2) Oligo-sets (G-T) in 20% denaturing gels Lanes 1-8: oligos A_8, GT_7, G_2T_6, G_3T_5, G_4T_4, G_5T_3, G_5TGT and G_5TG_2. All the oligo bands were visible and became stronger and stronger as base G increased. (G3) Oligo-set (T-C) in 20% denaturing gels. Lanes 1-8: oligos A_8, TC_7, T_2C_6, T_3C_5, T_4C_4, T_5C_3, T_6C_2 and T_7C. The bands of oligos T_2C_6, T_3C_5, T_4C_4, T_5C_3 and T_6C_2 were very weak. (H), (I) and (J) Oligo-sets (G-T) and (T-C) in 25%, 30% and 40% denaturing gels, respectively. Lanes 1-12: oligos A_8, GT_7, G_2T_6, G_3T_5, G_4T_4, G_5T_3, G_5TGT, G_5TG_2, T_3C_5, T_2C_6, TC_7 and C_7. In (H1), (I1) and (J1), oligo G_5TG_2 could be silver-stained and the other oligo bands of oligo-sets (G-T) and (T-C) were hazy or invisible. In (H2), (I2) and (J2), all the oligos of oligo-set (G-T) could be stained with SGRGS and the oligo bands became stronger and stronger as base G increased. The bands of oligos T_3C_5, T_2C_6, TC_7 and C_7 were weak. (K), (L), (M), (N) and (O) Oligo-set (T-G) in 15%, 20%, 25%, 30% and 40% denaturing gels, respectively. Lanes 1-11 of (K1), (K2), (O1) and (O2): oligos A_8, C_8, G_5, T_8, T_7G, T_6G_2, T_5G_3, T_4G_4, T_3G_5, $TGTG_5$ and G_2TG_5. Lanes 1-12 of (L1), (L2), (M1), (M2), (N1) and (N2): oligos A_8, C_8, T_6, T_7, T_8, T_7G, T_6G_2, T_5G_3, T_4G_4, T_3G_5, $TGTG_5$ and G_2TG_5. The oligo bands of oligo-sets (T-G) in (K1), (L1), (M1), (N1) and (O1) were hazy or invisible. The oligo bands of oligo-sets (T-G) in (K2), (L2), (M2), (N2) and (O2) were visible and became stronger and stronger as base G increase. Note: We are sorry

that some DNA bands shown are bad. The reasons why these DNA bands are bad have been mentioned in the figure legend of Fig 2.
