# Supplementary material for: Silver and Cyanine Staining of Oligonucleotides in Polyacrylamide Gel
Source: PLoS One. 2015 Dec 9;10(12):e0144422. doi: 10.1371/journal.pone.0144422 (PMC4674134; doi:10.1371/journal.pone.0144422)
Supplement: S3 Fig — (PDF) [file pone.0144422.s003.pdf]

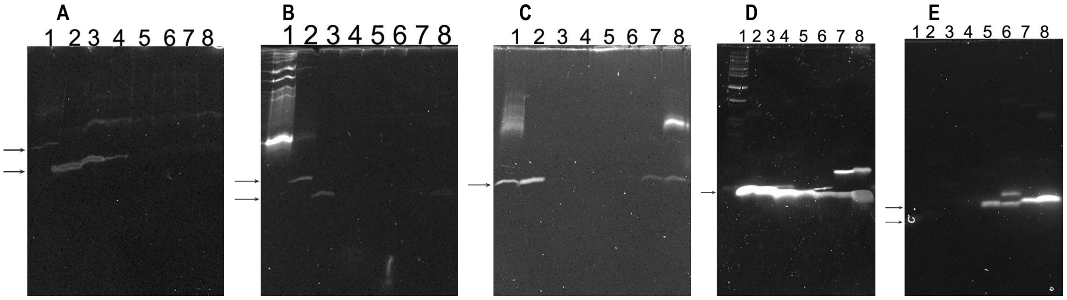

S3 Fig. Oligo-sets (A-C), (C-A) and (G-C) in denaturing PAGE gels stained with GDD and SGRGS. DNA markers were DNA marker 1, oligos A_{11}, A_8 and C_9. The arrows indicate the specific oligo bands. (A) Oligo-set (A-C) in 35% gel stained with GDD. Lanes 1-8: Oligos A_{11}, AC_7, A_2C_6, A_3C_5, A_4C_4, A_5C_3, A_6C_2 and A_7C. (B) Oligo-set (C-A) in 30% gel stained with GDD. Lane 1-8: DNA marker 1, oligos A_{11}, CA_7, C_2A_6, C_3A_5, C_4A_4, C_5A_3 and C_6A_2. (C) Oligo-set (C-A) in 30% gel stained with SGRGS. Lanes 1-8: Oligos A_8, CA_7, C_2A_6, C_3A_5, C_4A_4, C_5A_3, C_6A_2 and C_7A. (D) Oligo-set (G-C) in 15% gel stained with GDD. Lanes 1-8: DNA marker 1, oligos G_5CG_2, G_5C_2G, G_5C_3, G_4C_4, G_3C_5, G_2C_6 and GC_7. (E) Oligo-set (G-C) in 15% gel stained with SGRGS. Lanes 1-8: oligos C_9, GC_7, G_2C_6, G_3C_5, G_4C_4, G_5C_3, G_5C_2G and G_5CG_2. Note: We are sorry that some DNA bands shown are bad. The reasons why these DNA bands are bad have been mentioned in the figure legend of Fig 2.
