# Supplementary material for: Silver and Cyanine Staining of Oligonucleotides in Polyacrylamide Gel
Source: PLoS One. 2015 Dec 9;10(12):e0144422. doi: 10.1371/journal.pone.0144422 (PMC4674134; doi:10.1371/journal.pone.0144422)
Supplement: S4 Fig — (PDF) [file pone.0144422.s004.pdf]

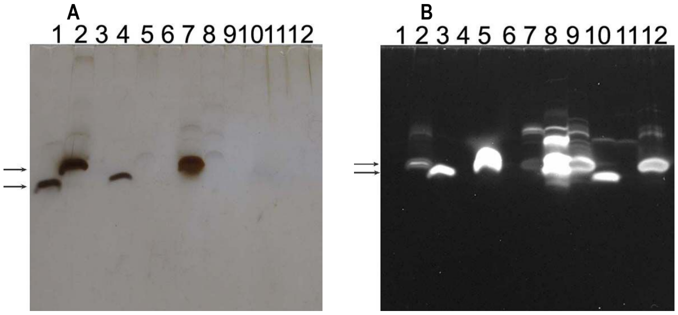

S4 Fig. Oligos A_4C_4, A_4G_4, A_4T_4, C_4A_4, C_4G_4, C_4T_4, G_3AGA_3, G_4C_4, G_4T_4, T_4A_4, T_4C_4 and T_4G_4 in 30% denaturing PAGE gel. DNA markers were oligos A_4C_4, A_4G_4 and A_4T_4.The arrows indicate the specific oligo bands. A and B were stained with silver and SGRGS, respectively. Lanes 1-12: oligos A_4C_4, A_4G_4, A_4T_4, C_4A_4, C_4G_4, C_4T_4, G_3AGA_3, G_4C_4, G_4T_4, T_4A_4, T_4C_4 and T_4G_4. Oligos C_4T_4, G_4T_4, T_4C_4 and T_4G_4 could not be silver-stained. The bands of oligos A_4T_4, T_4A_4, C_4G_4 and G_4C_4 silver-stained were weak or very weak. The bands of oligos A_4C_4, C_4A_4, C_4T_4 and T_4C_4 stained with SGRGS were invisible. Note: We are sorry that some DNA bands shown are bad. The reasons why these DNA bands are bad have been mentioned in the figure legend of Fig 2.
