# Supplementary material for: Silver and Cyanine Staining of Oligonucleotides in Polyacrylamide Gel
Source: PLoS One. 2015 Dec 9;10(12):e0144422. doi: 10.1371/journal.pone.0144422 (PMC4674134; doi:10.1371/journal.pone.0144422)
Supplement: S5 Fig — (PDF) [file pone.0144422.s005.pdf]

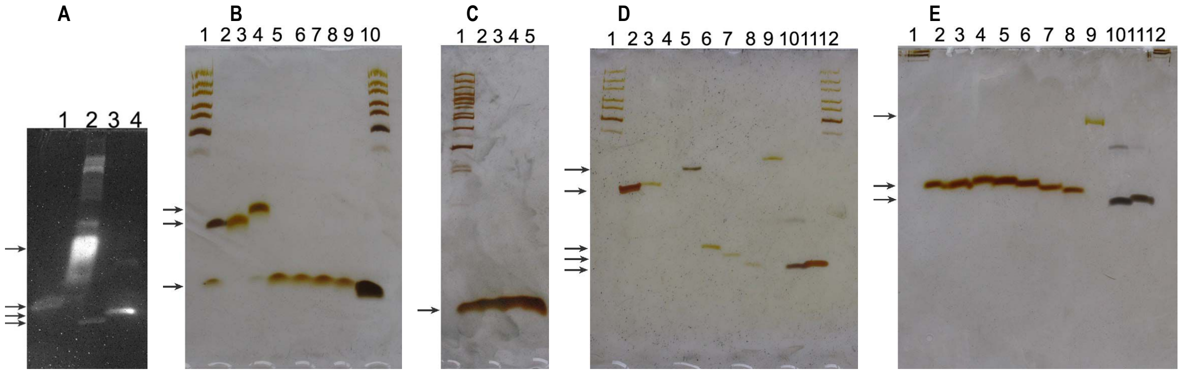

S5 Fig. Oligos T_9, G_4AG_4, C_9, A_9 and oligo-set (A-G) in different PAGE gel. The arrows indicate the specific oligo bands. (A) Oligos T_9, G_4AG_4, C_9, A_9 in 30% non-denaturing PAGE gel stained with SGRGS. Gel buffer: 1 × TBE. Tank buffer: 0.5 × TBE. Acrylamide/bisacrylamide = 19/1. Loading buffer was 6 × glycerol DNA loading buffer (Sangon, China). Electrophoresis was run at room temperature, 600V for 4 hrs. Lanes 1-4: Oligos T_9 (0.3 μg), G_4AG_4 (1 μg), C_9 (1 μg) and A_9 (0.056 μg). The oligos were not denatured at 95℃ before loading to the gel. DNA marker was oligo A_9. (B) and (C) 10-nt long oligo-set (A-G) in 15% denaturing PAGE gel silver-stained. Gel and Tank buffer: 0.5 × TBE. 7 M urea, Acrylamide/bisacrylamide = 29/1. Electrophoresis was run at room temperature, 200V for 1 hr. (B) Loading buffer was 6 × glycerol DNA loading buffer. The loading amount of each oligo was 1μg which was not denatured at 95℃ before loading to the gel. Lanes 1-10: DNA marker 1, oligos $(AG_3)_2AG$, $A_2G_3AG_4$, $A_3G_3AG_3$, $A_4G_2AG_3$, A_5GAG_3, A_7G_3, A_8G_2, A_9G and DNA marker 1. (C) loading buffer was formamide loading dye. The loading amount of each oligo was 1μg which was denatured at 95℃. Lanes 1-5: DNA marker 1, oligos $(AG_3)_2AG$, $A_2G_3AG_4$, $A_3G_3AG_3$ and $A_4G_2AG_3$. (D) 8-nt long oligo-set (A-G) in 20% non-denaturing PAGE gel silver-stained. Gel and Tank buffer: 0.5 × TBE. Acrylamide/bisacrylamide = 29/1. The gel contained 3% glycerol. Loading buffer was 6 × glycerol DNA loading buffer. The loading

amount of each oligo was 1μg which was not denatured at 95℃. Electrophoresis was run at room temperature, 200V for 1.7 hrs. Lanes 1-12: DNA marker 1, oligos $(AG_3)_2$, $A_2G_3AG_2$, A_3G_5, A_4G_4, A_5G_3, A_6G_2, A_7G, $A_{11}T_{11}$, C_7A, C_7G and DNA marker 1. (E) 8-nt long oligo-set (A-G) in 24% denaturing PAGE gel silver-stained. Gel and Tank buffer: 0.5 × TBE. 7 M urea, Acrylamide/bisacrylamide = 29/1. Loading buffer was formamide loading dye. The loading amount of each oligo was 1μg which was denatured at 95℃. Electrophoresis was run at room temperature, 200V for 2 hrs. Lanes 1-12: The same as those in (D). Note: We are sorry that some DNA bands shown are bad. The reasons why these DNA bands are bad have been mentioned in the figure legend of Fig 2.
