# Supplementary material for: Silver and Cyanine Staining of Oligonucleotides in Polyacrylamide Gel
Source: PLoS One. 2015 Dec 9;10(12):e0144422. doi: 10.1371/journal.pone.0144422 (PMC4674134; doi:10.1371/journal.pone.0144422)
Supplement: S6 Fig — (PDF) [file pone.0144422.s006.pdf]

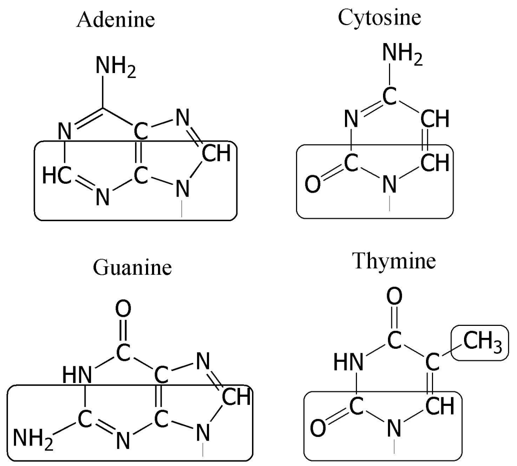

S6 Fig. A schematic diagram of the atoms which can affect the width of the cross sections of bases A, C, G and T that are vertical to the glycosidic bond between the base and the deoxyribose. The atoms in the rectangles are the atoms which can affect the width of the cross sections of bases A, C, G and T.
