# Supplementary material for: Silver and Cyanine Staining of Oligonucleotides in Polyacrylamide Gel
Source: PLoS One. 2015 Dec 9;10(12):e0144422. doi: 10.1371/journal.pone.0144422 (PMC4674134; doi:10.1371/journal.pone.0144422)
Supplement: S1 Table — (PDF) [file pone.0144422.s007.pdf]

S1 Table. The sequences of synthesized oligos

Oligo category	Length (nt)	Oligos	Sequences (5'-3')
Oligo (dA)	5	A_5	AAAAA
	6	A_6	AAAAAA
	7	A_7	AAAAAAA
	8	A_8	AAAAAAAA
	9	A_9	AAAAAAAAA
Oligo (dC)	5	C_5	CCCCC
	6	C_6	CCCCCC
	7	C_7	CCCCCCC
	8	C_8	CCCCCCCC
	9	C_9	CCCCCCCCC
Oligo (dG)	5	G_5	GGGGG
Oligo (dT)	5	T_5	TTTTT
	6	T_6	TTTTTT
	7	T_7	TTTTTTT
	8	T_8	TTTTTTTT
	9	T_9	TTTTTTTTT
	10	T_{10}	TTTTTTTTTT
	14	T_{14}	TTTTTTTTTTTTTT
	29	T_{29}	TTTTTTTTTTTTTTTTTTTTTTTTTTTTT
	31	T_{31}	TTTTTTTTTTTTTTTTTTTTTTTTTTTTTTT
	40	T_{40}	TTTTTTTTTTTTTTTTTTTTTTTTTTTTTTTTTTTTTTTT
	45	T_{45}	TTTTTTTTTTTTTTTTTTTTTTTTTTTTTTTTTTTTTTTTTTTTT
	50	T_{50}	TTTTTTTTTTTTTTTTTTTTTTTTTTTTTTTTTTTTTTTTTTTTTTTTTT
	59	T_{59}	TTTTTTTTTTTTTTTTTTTTTTTTTTTTTTTTTTTTTTTTTTTTTTTTTTTTTTTTTTT
Oligo-set (A-C)	8	AC_7	ACCCCCCC
	8	A_2C_6	AACCCCCC
	8	A_3C_5	AAACCCCC
	8	A_4C_4	AAAACCCC
	8	A_5C_3	AAAAACCC
	8	A_6C_2	AAAAAACC
	8	A_7C	AAAAAAAC
Oligo-set (A-G)	8	$(AG_3)_2$	AGGGAGGG
	8	$A_2G_3AG_2$	AAGGGAGG
	8	A_3G_5	AAAGGGGG
	8	A_4G_4	AAAAGGGG
	8	A_5G_3	AAAAAGGG
	8	A_6G_2	AAAAAAGG
	8	A_7G	AAAAAAAG
Oligo-set (A-T)	8	AT_7	ATTTTTTT
	8	A_2T_6	AATTTTTT
	8	A_3T_5	AAATTTTT
	8	A_4T_4	AAAATTTT
	8	A_5T_3	AAAAATTT
	8	A_6T_2	AAAAAATT
	8	A_7T	AAAAAAAT
	10	AT_9	ATTTTTTTTT
	10	A_2T_8	AATTTTTTTT
	10	A_3T_7	AAATTTTTTT
	10	A_4T_6	AAAATTTTTT
	10	A_5T_5	AAAAATTTTT
	10	A_6T_4	AAAAAATTTT
	10	A_7T_3	AAAAAAATTT
	10	A_8T_2	AAAAAAAATT
	10	A_9T	AAAAAAAAAT
Oligo-set (C-A)	8	CA_7	CAAAAAAA
	8	C_2A_6	CCAAAAAA
	8	C_3A_5	CCCAAAAA
	8	C_4A_4	CCCCAAAA
	8	C_5A_3	CCCCCAAA
	8	C_6A_2	CCCCCCAA
	8	C_7A	CCCCCCCA
Oligo-set (C-G)	8	$(CG_3)_2$	CGGGCGGG
	8	$CGCG_5$	CGCGGGGG
	8	C_3G_5	CCCGGGGG
	8	C_4G_4	CCCCGGGG
	8	C_5G_3	CCCCCGGG

	8	C_6G_2	CCCCCCGG
	8	C_7G	CCCCCCCG
Oligo-set (C-T)	8	CT_7	CTTTTTTT
	8	C_2T_6	CCTTTTTT
	8	C_3T_5	CCCTTTTT
	8	C_4T_4	CCCCTTTT
	8	C_5T_3	CCCCCTTT
	8	C_6T_2	CCCCCCTT
	8	C_7T	CCCCCCCT
Oligo-set (G-A)	8	GA_7	GAAAAAAA
	8	G_2A_6	GGAAAAAA
	8	G_3A_5	GGGAAAAA
	8	G_3AGA_3	GGGAGAAA
	8	$G_3AG_2A_2$	GGGAGGAA
	8	$(G_3A)_2$	GGGAGGGA
	8	G_3AG_4	GGGAGGGG
	8	G_5AG_2	GGGGGAGG
Oligo-set (G-C)	8	GC_7	GCCCCCCC
	8	G_2C_6	GGCCCCCC
	8	G_3C_5	GGGCCCCC
	8	G_4C_4	GGGGCCCC
	8	G_5C_3	GGGGGCCC
	8	G_5C_2G	GGGGGCCG
	8	G_5CG_2	GGGGGCGG
Oligo-set (G-T)	8	GT_7	GTTTTTTT
	8	G_2T_6	GGTTTTTT
	8	G_3T_5	GGGTTTTT
	8	G_4T_4	GGGGTTTT
	8	G_5T_3	GGGGGTTT
	8	G_5TGT	GGGGGTGT
	8	G_5TG_2	GGGGGTGG
	8	G_2TG_5	GGTGGGGG
Oligo-set (T-A)	8	TA_7	TAAAAAAA
	8	T_2A_6	TTAAAAAA
	8	T_3A_5	TTTAAAAA
	8	T_4A_4	TTTTAAAA
	8	T_5A_3	TTTTTAAA
	8	T_6A_2	TTTTTTAA
	8	T_7A	TTTTTTTA
Oligo-set (T-C)	8	TC_7	TCCCCCCC
	8	T_2C_6	TTCCCCCC
	8	T_3C_5	TTTCCCCC
	8	T_4C_4	TTTTCCCC
	8	T_5C_3	TTTTTCCC
	8	T_6C_2	TTTTTTCC
	8	T_7C	TTTTTTTC
Oligo-set (T-G)	8	$TGTG_5$	TGTGGGGG
	8	T_3G_5	TTTGGGGG
	8	T_4G_4	TTTTGGGG
	8	T_5G_3	TTTTTGGG
	8	T_6G_2	TTTTTTGG
	8	T_7G	TTTTTTTG
Others	9	$(ACG)_3$	ACGACGACG
	8	$(ACGT)_2$	ACGTACGT
	12	$(A_5T)_2$	AAAAATAAAAAT
	27	A_7T_{20}	AAAAAAATTTTTTTTTTTTTTTTTTTT
	27	A_8T_{19}	AAAAAAAATTTTTTTTTTTTTTTTTTT
	27	A_9T_{18}	AAAAAAAAATTTTTTTTTTTTTTTTTT
	16	$A_{10}T_6$	AAAAAAAAAATTTTTT
	18	$A_{10}T_8$	AAAAAAAAAATTTTTTTT
	20	$A_{10}T_{10}$	AAAAAAAAAATTTTTTTTTT
	22	$A_{11}T_{11}$	AAAAAAAAAAATTTTTTTTTTT
	24	$A_{12}T_{12}$	AAAAAAAAAAAATTTTTTTTTTTT
	24	$(TA)_{12}$	TATATATATATATATATATATATA
	24	$A_{14}T_{10}$	AAAAAAAAAAAAAATTTTTTTTTT
	30	$A_{15}T_{15}$	AAAAAAAAAAAAAAATTTTTTTTTTTTTTT
	27	$A_{17}T_{10}$	AAAAAAAAAAAAAAAAATTTTTTTTTT
	40	$A_{20}T_{20}$	AAAAAAAAAAAAAAAAAAAATTTTTTTTTTTTTTTTTTTT
