# Supplementary material for: Silver and Cyanine Staining of Oligonucleotides in Polyacrylamide Gel
Source: PLoS One. 2015 Dec 9;10(12):e0144422. doi: 10.1371/journal.pone.0144422 (PMC4674134; doi:10.1371/journal.pone.0144422)
Supplement: S2 Table — (PDF) [file pone.0144422.s008.pdf]

S2 Table. Δ IntDen of oligo A_{11} stained with Silver and SGRGS

LAO (μg)	0.01	0.02	0.03	0.04	0.08	0.1	0.2	0.4	0.5	0.75	1
Δ Int(Si/Sg)	-5503/544	-8187/721	-66995/1683	-128901/2780	-135140/4003	-153437/8094	-180754/12828	-327602/17515	-389158/21497	-398754/23414	-478697/24489

LAO = the loading amount of oligo A_{11}. Si = Silver staining. Sg = SGRGS staining.
